# Down But Not Out: Vasectomy Is Faring Poorly Almost Everywhere—We Can Do Better To Make It A True Method Option

**DOI:** 10.9745/GHSP-D-22-00369

**Published:** 2023-02-28

**Authors:** Roy Jacobstein, Scott Radloff, Farhad Khan, Kathryn Mimno, Manoj Pal, Jennifer Snell, Renae Stafford, Cheick Touré, Vandana Tripathi

**Affiliations:** a Consultant, Cary, NC, USA.; b The Bill & Melinda Gates Institute for Population and Reproductive Health, Johns Hopkins University, Baltimore, MD, USA.; c MOMENTUM Safe Surgery in Family Planning and Obstetrics, EngenderHealth, Washington, DC, USA.; d MOMENTUM Safe Surgery in Family Planning and Obstetrics, IntraHealth International, Chapel Hill, NC, USA.; e MOMENTUM Safe Surgery in Family Planning and Obstetrics, EngenderHealth, New Delhi, India.; f MOMENTUM Safe Surgery in Family Planning and Obstetrics, IntraHealth International, Bamako, Mali.

## Abstract

**Introduction:**

Demand for vasectomy—1 of 2 contraceptive methods for men—has been low, with deep-seated myths, misconceptions, and provider bias against it widespread. Programmatic attention and donor funding have been limited and sporadic.

**Methods:**

We analyzed vasectomy use in 84 low- and middle-income countries (LMICs) plus the 11 high-income countries with vasectomy prevalence above 1%. These 95 countries comprise 90% of the world’s population. Data come from United Nations survey compilations, population estimates, and gender inequality rankings. We also reviewed recent articles on vasectomy and analyses of chronic challenges to vasectomy service provision.

**Results:**

Vasectomy use is 61% lower now than 2 decades ago. Of 922 million women using contraception worldwide, 17 million rely on vasectomy—27 million fewer than in 2001. In contrast, 219 million women use tubectomy—8 million more than in 2001. Of 84 LMICs, 7 report vasectomy prevalence above 2%. In 56 LMICs, no more than 1 in 1,000 women relies on vasectomy. Female-to-male disparities in permanent method use widened globally, from 5:1 to 13:1, and are much higher in some regions and countries (e.g., 76:1 in India). Countries with the highest vasectomy prevalence are among those with the highest gender equality and vice versa.

**Conclusion:**

Vasectomy use is surprisingly low globally and declining. Use remains negligible in almost all LMICs, reflecting low demand and program priority. For vasectomy to become an accessible, rights-based option, program efforts need to be holistic, ensuring an enabling environment while coordinating demand- and service-focused efforts. Vasectomy champions at all levels should be supported on a sustained basis. On the demand side, harnessing mass and social media to increase accurate knowledge and normalize vasectomy as a method and service will be particularly valuable. Evidence from Bolivia suggests relatively few trained providers and procedures could result in a country’s attaining 1% vasectomy prevalence.

## Abstract (precis)

Contraceptive use worldwide increased by 188 million users in the past 20 years. Yet the number of vasectomy users fell by 27 million, a 61% decline. Almost all LMICs report negligible vasectomy use. We can do better to make it an accessible rights-based option.

## Teaser

**Key Findings:**

- Vasectomy use has markedly declined globally, to only 39% of what it was in 2001, even as overall contraceptive use has risen in most countries. Almost all low- and middle-income countries (LMICs) report negligible to zero vasectomy prevalence. In 56 LMICs, no more than 1 in 1,000 women relies on vasectomy. Multiple demand-side and supply-side challenges hinder vasectomy’s becoming an available program method.
- Only 7 countries, including 3 LMICs, registered increases in vasectomy prevalence between recent surveys. Seven LMICs—Bhutan, Brazil, Colombia, Costa Rica, Iran, Mexico, and Nepal—have achieved substantial vasectomy prevalence (above 2%).
- Countries with the lowest gender inequality are among those with the highest vasectomy prevalence, and vice versa. Female-to-male disparities in permanent method use have widened further in the past 20 years.

**Key Implications:**

For vasectomy to become a truly available, rights-based method option:

- Policymakers and donors should allot more time, attention, and priority to vasectomy; provide additive and sustained funding; and use metrics of success that focus on program inputs and progress toward longer-term goals, not immediate vasectomy uptake.
- Program managers should coordinate demand-side and supply-side interventions, promote constructive male engagement, identify and support vasectomy champions, and use a range of communication modalities to increase accurate knowledge of vasectomy.

## INTRODUCTION

Male sterilization (vasectomy) is 1 of only 2 modern contraceptive methods for men. Vasectomy, like female sterilization (hereafter in this article, tubectomy), is a safe, highly effective, permanent method (PM) of contraception.^1^^,^^2^ As PMs, vasectomy and tubectomy are suitable only for people intending to limit further births. Both methods entail minor surgery and require skilled service providers and supportive health systems. All men are eligible for vasectomy. Vasectomy is safer, easier, and less time consuming to perform (10–20 minutes) than tubectomy. Tubectomy is immediately effective; however, vasectomy has a 3-month delay in taking full effect, requiring the use of an additional contraceptive method for the first 3 months post-vasectomy. Service programs need to ensure PMs are provided in a rights-based manner: chosen knowingly and voluntarily by clients who wish to limit further births, from a wide range of contraceptive options, and free of any pressure or coercion.^3^^,^^4^

There is longstanding international consensus on the importance of achieving gender equality (Sustainable Development Goal [SDG] 5)^5^; ensuring free, voluntary, and informed choice for people from a broad range of method options,^6^ including PMs^7^; and fostering greater constructive male involvement in family planning (FP), with men acting not only as supportive partners and advocates but also as users of contraception themselves.^8^ In 1954, the first national-level vasectomy program was launched in India.^9^ Despite vasectomy’s early programmatic introduction and positive method features, there has been low demand for vasectomy services, and deep-seated myths and misunderstandings about it are widespread. Programmatic attention to vasectomy and donor funding for it has been limited and sporadic, as FP program efforts and service provision have been largely oriented to women and their own contraceptive use.

Despite vasectomy’s early programmatic introduction and positive method features, there has been low demand for vasectomy services.

In this article, we seek to deepen the international FP community’s focus on vasectomy, underscoring its tenuous situation. We highlight trends in vasectomy use by comparing current vasectomy prevalence levels to those of approximately a decade earlier in 95 countries, 84 of them low- and middle-income countries (LMICs). These countries comprise around 90% of the world’s population, including 18 of its 20 most populous countries.^10^ We also compare and contrast the situation of vasectomy and tubectomy. We note chronic programmatic challenges yet aim to make a cogent advocacy case for greater attention to and investment in vasectomy. In addition, we suggest how to improve programming for vasectomy so that it becomes a regularly available and accessible, rights-based method option for people in LMICs, as it is in some high-income countries (HICs). Finally, we hope this article may serve as a convenient and comprehensive repository of the latest available information on recent trends and current prevalence of use of vasectomy, as well as tubectomy, in almost all countries in Africa, Asia, and Latin America and the Caribbean (LAC).

## METHODS

To assess vasectomy’s current prevalence and method share, trends across several decades, and comparisons of its use to that of tubectomy, we drew from 2 resources recently produced by the United Nations Department of Social Affairs (UNDESA): “World Contraceptive Use 2022” (“UNDESA 2022”)^11^; and “Contraceptive Use by Method 2019: Data Booklet” (“UNDESA 2019”).^12^ UNDESA 2022 provides the latest compilation by the United Nations (UN) of population-based surveys of estimated contraceptive use in countries for married or in-union women of reproductive age (MWRA). It includes 1,404 surveys of contraceptive use from the 1970s onward that pertain to 197 countries and member states. This resource is continually updated as new surveys become available, with the most recent data (at the time of this writing) updated through April 2022. We relied on data from this source in analyzing country-level use of vasectomy and trends in its prevalence.

We also relied on UNDESA 2019’s Annex Table of Key Indicators for estimates of population totals of all women of reproductive age (WRA) in various regions, subregions, and development groupings. These totals, not provided in UNDESA 2022, are needed to calculate numbers of method users. Using data from these 2 sources, we calculated the number of PM users according to the formula: estimated prevalence x estimated population. Each method’s share of the modern contraceptive method mix was calculated according to the formula: specific PM prevalence rate/modern contraceptive prevalence rate (MCPR). Female-to-male (F:M) ratios of PM use were calculated according to the formula: tubectomy prevalence/vasectomy prevalence. In this article, we speak of “prevalence” and “number(s) of users” rather than “estimated prevalence” and “estimated number(s) of users.”

Selection criteria for LMICs’ inclusion in our analysis were their having: (1) a population of WRA above 1 million (in UNDESA 2019), equating to a population above 4 million people; and (2) at least 2 surveys, including a most recent survey of contraceptive use conducted after 2010 (in UNDESA 2022). We included every LMIC from Africa, Asia, and LAC meeting these criteria, as well as Bhutan and Qatar, LMICs with populations below 1 million WRA but vasectomy prevalence above 1%. Surveys from 84 LMICs met our selection criteria: 32 from sub-Saharan Africa (18 in Western Africa and Middle Africa and 14 in Eastern Africa and Southern Africa, as designated by UNDESA); 15 from Northern Africa, Central Asia, and Western Asia; 20 from Southern Asia, South-Eastern Asia, and Eastern Asia; and 17 from LAC. We also included 11 HICs, the only HICs with surveys in UNDESA 2022 that indicate a vasectomy prevalence of 1% or higher. (We limited analysis of vasectomy use in HICs in this manner to maintain our article’s primary focus on LMICs, while also affording meaningful country comparisons). For countries that met these criteria, we selected their most recent survey and a similar survey conducted 8–12 years earlier (if possible), enabling assessment of trends over a most recent decade. In 71 of the LMICs, the most recent survey of contraceptive use was conducted between 2015 and 2022.

Survey data from countries that met our study criteria, including their MCPR, vasectomy prevalence, vasectomy method share, and tubectomy prevalence, are presented in Table 1 for the 20 countries (including 9 LMICs) with the highest vasectomy prevalence in the world (above 1%). Similar data for 75 LMICs with vasectomy prevalence below 1% are grouped by regions in 5 tables in the Supplement. Numerical values for vasectomy prevalence in both the earlier and latest survey are provided in the UNDESA 2022 compilation for 44 LMICs, while 40 LMICs had no value for vasectomy prevalence indicated, including in the latest surveys from 26 LMICs. For surveys that did not indicate a value for vasectomy prevalence, typically due to the very low number of surveyed women indicating reliance on vasectomy, we assigned a value of zero in our analysis and indicated this absence in the Supplement tables with dashes.

**TABLE 1.** Vasectomy Prevalence, Vasectomy Method Share, and Tubectomy Prevalence in the Only 20 Countries in the World With Vasectomy Prevalence Above 1%^a^

**Country**	**Survey End Year**	**Age Group, Years**	**MCPR,** **%**	**Vasectomy Prevalence, %**	**Vasectomy Method Share,**^b^ **%**	**Tubectomy** **Prevalence, %**
South Korea	2009	15–44	66.4	16.8	25.3	5.9
	2000	15–44	71.5	13.0	18.2	18.5
Australia	2016	18–44	64.7	14.0	21.6	5.1
	2006	18–44	63.1	15.2	24.1	7.0
Bhutan^c^	2010	15–49	65.4	12.6	19.3	7.1
	2000	15–49	30.7	13.6	44.3	3.1
United States	2019	15–49	66.1	11.3	17.1	19.0
	2010	15–44	70.4	11.0	15.6	22.1
New Zealand	2015	16–49	74.7	10.1	13.5	5.3
	1995	20–49	72.3	19.5	27.0	14.6
United Kingdom	2012	16–49	71.1	9.8	13.8	5.9
	2002	16–49	82.0	19.0	23.2	12.0
Belgium	2010	18–49	69.1	8.4	12.2	8.4
	1992	21–39	74.3	7.0	9.4	10.9
Canada	2006	15–49	85.0^d^	7.4	8.7	7.0
	2002	18–44	72.0^d^	22.0	30.6	11.0
Spain	2018	18–49	59.9	6.2	10.4	2.2
	2006	15–49	62.3	7.9	12.7	5.6
Netherlands	2013	18–45	70.0	6.0	8.6	3.0
	2003	18–45	73.0	10.0	13.7	4.0
Taiwan	2016	18–45	65.5	5.8	8.8	13.4
	2008	18–45	67.1	2.7	4.0	24.5
Costa Rica	2018	15–49	69.0	5.2	7.5	21.9
	2010	15–49	79.9	5.9	7.4	33.0
Brazil	2013	18–49	77.7	4.2	5.4	21.4
	2007	15–49	77.1	5.1	6.6	29.1
Colombia	2016	15–49	75.9	3.6	4.7	35.0
	2005	15–49	68.2	1.8	2.6	31.2
Nepal	2019	15–49	44.2	3.5	7.9	12.9
	2011	15–49	43.2	7.8	18.1	15.2
Iran	2011	15–49	57.0	2.8	4.9	14.2
	2002	15–49	58.9	2.3	3.9	15.2
Mexico	2018	15–49	69.8	2.3	3.3	37.1
	2009	15–49	67.4	2.2	3.3	36.3
China	2017	15–49	80.5	1.4	1.7	18.3
	2006	15–49	84.0	4.5	5.4	28.7
Sweden	2017	15–49	68.1	1.1	1.6	1.4
	2013	15**–**49	68.8	**--**	**--**	1.9
Qatar^c^	2012	15–49	34.4	1.1	3.2	1.4
	1998	15–49	32.3	**--**	**--**	4.1

Abbreviations: MCPR, modern contraceptive prevalence rate; MWRA, married or in-union women of reproductive age; WRA, women of reproductive age.

a Data are for women married or in union (MWRA).

b Vasectomy’s method share is its proportion of use among users of modern contraception.

c Although not meeting study criteria for WRA population size, Bhutan and Qatar are included in table because they are less populous low- and middle-income countries with vasectomy prevalence above 1%.

d Any-method prevalence, including traditional methods.

Source: UN Department of Economic and Social Affairs.^11^

We note that the 2 survey-based sources we relied on used different denominators in their calculations. However, because vasectomy is almost always relied on by married or in-union women, all or nearly all “vasectomy users” in surveys (i.e., the women relying on a male partner's vasectomy) are included in the numerator regardless of whether WRA or MWRA is the denominator in calculations of vasectomy prevalence. We also point out that a decline in vasectomy prevalence between 2 survey dates implies that the number of new vasectomy users/procedures performed in a country has not offset the number of women who are no longer of reproductive age (aged 49 years in almost all the surveys), and/or has not kept pace with population growth during the inter-survey interval. Conversely, due to population growth, an identical vasectomy prevalence at a later survey date equates to more vasectomy users.

Estimates of the number of vasectomy procedures performed in a country are not easily obtainable from population-based surveys but rather are typically ascertained via service statistics. However, based on age-specific population estimates for LMICs excluding China, age-specific vasectomy rates in the United States,^13^^,^^14^ and recent experience with vasectomy service provision in Bolivia (discussed later), we constructed a calculator for estimating what vasectomy prevalence rates might be reached, given a specified number of vasectomy clients being served annually over a 15-year period. This calculator takes account of population growth, mortality, and a woman’s aging out of a survey’s upper limit for reproductive age, which all affect both the numerator and the denominator in the calculation of vasectomy prevalence rates. We also note that the calculations we generate are provided for illustrative purposes only, not for setting targets, which is always an unacceptable programming practice.

To consider vasectomy use and gender equality, we compared country rankings on vasectomy prevalence, which we generated, with country rankings on Gender Inequality Index (GII) generated by the United Nations Development Programme (UNDP) in 2018,^15^ adjusted by the authors according to study criteria. The UNDP ranks 162 countries on gender (in)equality, a component of its Human Development Index. GII is a composite metric using 3 dimensions: reproductive health (RH), empowerment, and labor markets. Included in RH are the maternal mortality ratio (MMR, SDG 3.1) and the adolescent birth rate (SDG 3.7) but no measure of contraceptive use. Low numerical ranking of a country on GII indicates lower inequality (or higher equality) between men and women.

We adjusted the UNDP's GII country rankings by removing from consideration of rank any country not meeting our study selection criteria (e.g., for small population size or not reporting on recent vasectomy prevalence). We then renumbered the remaining countries’ GII rankings according to these adjustments (Table 2). We also included France in our analysis in consideration of its very high ranking on gender equality, although it has a vasectomy prevalence below our cutoff level (for HICs) of 1%. Taiwan, an HIC with substantial vasectomy prevalence, is also included in Table 2, although we are unable to consider correspondence to gender equality because no GII information for Taiwan is provided by the UNDP. Four countries in Northern or Western Europe with very low gender inequality, very high MCPR, and substantial vasectomy use historically are excluded from our adjusted country rankings of GII because they either did not have a survey beyond the mid-1990s (Norway and Denmark) or do not indicate method-specific contraceptive prevalence figures for PMs (Switzerland and Finland).

**TABLE 2.** Country Rankings on Vasectomy Prevalence and Adjusted Rankings on Gender Inequality Index in the 21 Countries With the World’s Highest Vasectomy Prevalence

**Country**	**Survey Year**	**Vasectomy Prevalence,**^a^ **%**	**Tubectomy Prevalence,**^a^ **%**	**M:F Ratio of PM Prevalence** ^a^	**Ranking on Vasectomy Prevalence** ^a^	**UN Country Ranking on GII, Adjusted** ^b^
South Korea	2009	16.8	5.9	2.80:1	1	5
Australia	2016	14.0	5.1	2.70:1	2	8
Bhutan	2010	12.6	7.1	1.80:1	3	38
United States	2019	11.3	19.0	0.60:1	4	12
New Zealand	2015	10.1	5.3	1.90:1	5	10
United Kingdom	2012	9.8	5.9	1.70:1	6	9
Belgium	2010	8.4	8.4	1.00:1	7	3
Canada	2006	7.4	7.0	1.10:1	8	7
Spain	2018	6.2	2.2	2.80:1	9	6
Netherlands	2013	6.0	3.0	2.00:1	10	2
Taiwan	2016	5.8	13.4	0.40:1	11	^__c^
Costa Rica	2018	5.2	21.9	0.20:1	12	17
Brazil	2013	4.2	21.4	0.20:1	13	31
Colombia	2016	3.6	35.0	0.10:1	14	34
Nepal	2019	3.5	12.9	0.30:1	15	51
Iran	2011	2.8	14.2	0.20:1	16	54
Mexico	2018	2.3	37.1	0.06:1	17	24
China	2017	1.4	18.3	0.08:1	18	11
Sweden	2017	1.1	1.4	0.80:1	19	1
Qatar	2012	1.1	0.8	1.40:1	19	13
France	2008	0.8	3.8	0.20:1	21	4

Abbreviation: GII, Gender Inequality Index; M:F, male-to-female; MWRA, married or in-union women of reproductive age; PM, permanent method; UN, United Nations.

a Prevalence data are for women married or in union (MWRA).

b UN country rankings on GII are adjusted by authors, based on a subset of 91 countries that met study selection criteria of: (a) at least 1 million women of reproductive age and (b) 2 recent surveys fielded roughly 10 years apart that provide a value for vasectomy prevalence.

c No figure for Taiwan’s GII is provided in this UN source.

Source: For vasectomy prevalence values, UN Department of Economic and Social Affairs.^11^ For GII country ranking, United Nations Development Programme.^15^

**FIGURE 1:**
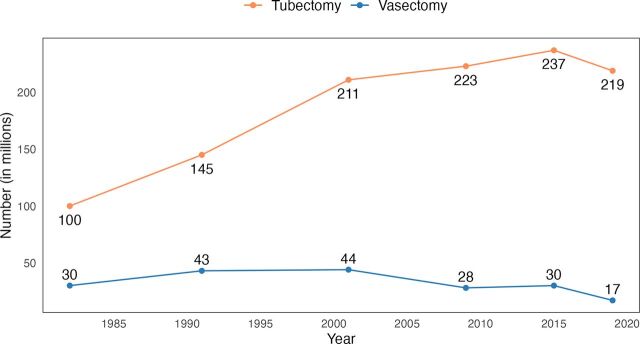
Trends in Global Use of Vasectomy and Tubectomy, 1982–2019 Data Sources**:** For 1982, 1991, and 2001, EngenderHealth 2002.^3^ For 2009, EngenderHealth 2014.^4^ For 2015 and 2019, UNDESA 2015,^17^ and UNDESA 2019.^12^

We also conducted a literature search on PubMed for articles with “vasectomy” or “male sterilization” in their title that appeared between 2012 and 2022 and concerned LMICs.^16^ We also searched 7 journals devoted to FP/RH or health more generally for mention of vasectomy during that past decade. We cite 8 articles that relate to vasectomy programming from 4 of these journals (*Contraception*; *Global Health: Science and Practice*; *Lancet Global Health*; *Studies in Family Planning*); no articles on vasectomy were identified in the other 3 journals (*Bulletin of the World Health Organization*; *International Perspectives on Sexual and Reproductive Health*; *Sexual and Reproductive Health Matters*). We also drew on EngenderHealth’s 2 comprehensive analyses of trends in PM use and chronic issues and challenges in programming for PM service provision, published in 2002 and 2014.^3^^,^^4^ Figure 1 updates a similar graph in EngenderHealth’s 2014 White Paper through 2019.

## FINDINGS

### Global and Regional Trends in Vasectomy Use

Global vasectomy use declined markedly between 2001 and 2019.^12^^,^^17^ Current vasectomy use is only 39% of what it was in 2001. According to our calculations from UNDESA 2019 figures, about 17 million women rely on (a male partner’s) vasectomy globally (Table 3), down from peaks of 43 million in 1991 and 44 million in 2001 (Figure 1). This decline has occurred in the face of trends that might have been expected to increase vasectomy use: the world’s population has increased 44% since 1991,^10^ use of modern contraception has risen substantially in most LMICs, and demand to limit further births now exceeds demand to space births among MWRA in all regions except Western Africa and Middle Africa.^18^ Global vasectomy prevalence is indicated in UNDESA 2019 to be 0.9% among the world’s 1.9 billion WRA, accounting for a 1.9% share of global contraceptive use (of any method, including traditional methods). The F:M ratio in global PM use has widened markedly, from 3:1 in 1982 and 1991 to 5:1 in 2001 and 13:1 in 2019.

**TABLE 3.** Vasectomy and Tubectomy Prevalence, Number of Users, Method Share, and Ratios of PM Use, in Selected UN-Designated Categories^a^

**Category**	**WRA,**^b^ **No.**	**CPR** **(All Methods), %**	**WRA Using Any Method,^c^ No.**	**PM** **Prevalence** ^c^		**Users** **of Each PM**^d^**, No.**		**Method Share** **of Each PM**^d^		**F:M** **(T:V) Ratio**^d^
				**T, %**	**V, %**	**T**	**V**	**T, %**	**V, %**	
World	1.901 billion	48.5	922 million	11.5	0.9	218.6 million	17.1 million	23.7	1.9	13:1
HICs	271 million	56.6	153 million	6.1	3.0	16.5 million	8.1 million	10.8	5.3	2:1
LMICs	1.63 billion	47.1	768 million	12.4	0.6	202.4 million	9.2 million	26.4	1.2	22:1
LMICs excluding China	1.287 billion	41.1	529 million	12.0	0.4	154.1 million	5.4 million	29.1	1.0	29:1
Sub-Saharan Africa	258 million	28.5	74 million	1.1	0.0	2.8 million	negligible	3.9	0.0	---
LAC	175 million	58.0	101 million	16.0	1.3	28 million	2.3 million	27.6	2.2	12:1
China	343 million	69.6	239 million	14.1	1.1	48.3 million	3.8 million	20.3	1.6	13:1
India	354 million	42.6	151 million	29.0	0.2	102.7 million	710 thousand	68.1	0.5	145:1^e^

Abbreviations: CPR, contraceptive prevalence rate; F:M, female-to-male; HIC, high-income country; LAC, Latin America and the Caribbean; LMIC, low- and middle-income country; PM, permanent method; T, tubectomy; UN, United Nations; UNDESA, United Nations Department of Economic and Social Affairs; V, vasectomy; WRA, women of reproductive age.

a Category of LMICs consolidated by authors, combining UNDESA categories of low-income countries and middle-income countries.

b Women aged 15–49 years.

c (Estimated) values for method prevalence and numbers of WRA taken from data source’s Annex Table of Key Indicators.

d Values for (estimated) numbers of PM users, respective method shares, and T-to-V (F:M, female-to-male) ratios calculated by authors.

e In UNDESA 2022, India’s modern CPR in its 2021 survey is 56.5%, tubectomy prevalence 37.9%, and vasectomy prevalence 0.3%, yielding an F:M ratio in PM use of 76:1.

Source: UN Department of Economic and Social Affairs.^12^

Global vasectomy use has declined markedly, to only 39% of what it was in 2001, despite trends that might have been expected to increase its use.

In the UNDESA grouping of LMICs excluding China, vasectomy prevalence is 0.5%. This equates to 5.4 million vasectomy users among a total of 529 million WRA using contraception and accounts for 1.2% of contraceptive use (method share). From a regional or subregional standpoint, vasectomy prevalence is highest in Oceania, Northern America, and Northern Europe and lowest—0.0%—in Northern Africa, sub-Saharan Africa, Central Asia, and Western Asia, regions that encompass many of the world’s least developed countries.

### Countries With Vasectomy Prevalence Above 1%

Twenty countries listed in UNDESA 2022 have vasectomy prevalence above 1%, with 12 having prevalence above 5% (Table 1).^11^ Nine of the 20 countries are LMICs, 7 of which have a vasectomy prevalence above 2%: Bhutan, Brazil, Colombia, Costa Rica, Iran, Mexico, and Nepal. Formerly but no longer classified as an LMIC, South Korea has the world’s highest vasectomy prevalence, 16.8%, and has maintained double-digit vasectomy prevalence since 1988. Four other countries also have double-digit vasectomy prevalence: Australia (14.0%), Bhutan (12.6%), United States (11.3%), and New Zealand (10.1%). Only 4 countries increased vasectomy prevalence by more than half a percentage point in a most recent decade: Belgium, Colombia, South Korea, and Taiwan. Vasectomy prevalence in Taiwan, also a former LMIC, increased by 115% in 8 years to 5.8% in 2016. Colombia’s vasectomy prevalence also doubled, from 1.8% in 2005 to 3.6% in 2016. Two LMICs with vasectomy prevalence above 5% in their earlier survey registered declines in their latest survey: Brazil, from 5.1% in 2007 to 4.2% in 2013, and Nepal by more than two-thirds (68%), from 7.8% in 2011 to 3.5% in 2019. Vasectomy prevalence declined by about half over the past 1–2 decades, from very high to still-substantial levels, in New Zealand and the United Kingdom. Before its most recent survey, the United Kingdom had registered double-digit vasectomy prevalence in 20 previous surveys between 1986 and 2012.

### Vasectomy’s Method Share in Countries With Vasectomy Prevalence Above 1%

In the 20 countries with vasectomy prevalence above 1%, vasectomy’s share of the method mix among MWRA ranges from less than 2% in Sweden and China to more than 17% in Australia, Bhutan, South Korea, and the United States (Table 1). Vasectomy’s method share is highest in South Korea, where 1 in every 4 MWRA using modern contraception relies on vasectomy. In the 7 countries with the highest vasectomy prevalence, vasectomy’s method share is in the double digits (i.e., at least 1 in 10 MWRA using modern contraception relies on vasectomy). Vasectomy’s method share increased in 7 countries: Belgium, Colombia, Costa Rica, Iran, South Korea, Taiwan, and the United States. It declined in 10 countries, substantially so in Bhutan, Canada, China, Nepal, Netherlands, New Zealand, and the United Kingdom. In the United Kingdom’s most recent survey from 2012 and its comparison survey from 2002, vasectomy’s method share was twice that of tubectomy, although method share declined by about half for both PMs. Vasectomy’s method share exceeded that of tubectomy in 17 of the United Kingdom’s 20 surveys from 1986 through 2012. Colombia and Taiwan had the highest percentage increases in vasectomy’s method share of any country, almost doubling in Colombia (81% increase) and more than doubling in Taiwan (122% increase).

### Prevalence and Method Share in LMICs With Vasectomy Prevalence Below 1%

Of the 84 LMICs that met our study selection criteria, 75 LMICs have current vasectomy prevalence below 1%, with 56 of them having a vasectomy prevalence of 0.1% or lower—i.e., in 56 LMICs, no more than 1 in 1,000 women rely on vasectomy. Thirty-nine LMICs have zero or no reported vasectomy prevalence. Vasectomy’s share of the method mix in all of these countries is concomitantly low, only exceeding 1% in Guatemala, Papua New Guinea, and South Africa.

In 56 LMICs, no more than 1 in 1,000 women rely on vasectomy.

From a regional standpoint, the only African countries with vasectomy prevalence and method share above 0.1% are Rwanda and South Africa (as well as Lesotho and Namibia, excluded from our study because of small population size.) In Southern Asia, which comprises 26% of the world’s population and where PM use to limit further births is a societal norm (as it is not in Africa),^12^ vasectomy prevalence and method share are also low and have trended downward. Vasectomy’s prevalence and method share have declined by two-thirds in Bangladesh and India and remain unmeasurable in Pakistan. In Eastern Asia and South-Eastern Asia, vasectomy prevalence is higher than 0.3% only in China (discussed later) and Papua New Guinea. Thailand’s vasectomy prevalence, once as high as 5.7% in 1987, was 0.3% in 2019. Overall, vasectomy prevalence has declined in every Asian LMIC except Iran, whose most recent survey was in 2011. In LAC, also a region where PM use is the norm, 13 of 17 countries have a vasectomy prevalence of 0.4% or lower, even though tubectomy prevalence is above 9% in every LAC country except Haiti and above 20% in 11 LAC countries.

### China and India: Despite Falling Prevalence, Still Main Contributors to Global Vasectomy Use

As it has been historically, China is by far the largest single contributor to global and LMIC vasectomy totals, accounting for more than 1 in 5 current vasectomy users (21%) worldwide. Around 3.8 million women relied on vasectomy in China in 2017, a decline of 6.1 million (69%) from the number of women who relied on a partner’s vasectomy a decade earlier. Vasectomy prevalence, which peaked in China in 1992 at 10.2%, declined to 4.5% prevalence in 2006, and declined to 1.4% prevalence in 2017 (as reported in UNDESA 2022)—a decline likely to continue.^19^ Vasectomy’s method share in China in 2017 was 1.7%, one-third of vasectomy’s method share a decade earlier and one-seventh of vasectomy’s peak method share of 12.2% in 1992.

India, the second-largest contributor to global and LMIC vasectomy totals, accounts for an additional 4% of global vasectomy use. Like China, India has also registered substantial and continuing declines in vasectomy’s use and method share. From a peak vasectomy prevalence of 3.5% in 1993 (9.6% method share), vasectomy prevalence in India declined more than 3-fold to 1.0% in 2006 and declined a further 3-fold to 0.3% in 2016 (0.6% method share).^11^ The combined decline in vasectomy use in China and India from 2006 to 2016–2017 amounts to around 6.8 million users.^12^ This decline accounts for 62% of the global decline in vasectomy use of 11 million from 2009 to 2019.^20^

### Comparison of Vasectomy and Tubectomy Use Globally and in LMICs

Whereas vasectomy use has declined by nearly two-thirds (61%) globally during the past 2 decades, from 44 million users to 17 million users, tubectomy use has ranged from 211 million to 237 million users the past 2 decades and was used by 8 million more women in 2019 than in 2001 (Figure 1). Tubectomy remains the world’s most widely-used contraceptive method, used by almost 1 in every 4 WRA using contraception. F:M disparities in use of the 2 PMs have continued to widen globally, from 3:1 in 1982 to 5:1 in 2001 to 13:1 in 2019. Furthermore, regional disparities in F:M use of PMs are often larger than global disparities. According to UNDESA 2019 figures, in every geographic region or UN developmental group category except HICs, tubectomy’s prevalence and method share exceed that of vasectomy by a factor of 12 or greater (Table 3).^12^ The F:M disparity in PM use in LMICs excluding China—where donors focus their priorities and financial assistance for FP—is 29:1, with 154 million WRA using tubectomy in these countries compared to fewer than 5.5 million relying on a male partner’s vasectomy. In LAC, with the world’s highest regional PM prevalence, tubectomy’s prevalence (16%) is 12 times greater than vasectomy’s prevalence (1.3%). Tubectomy’s method share in LAC is 55 times larger than vasectomy’s method share, though this disparity is beginning to fall in some LAC countries.

Female to male disparities in use of the 2 PMs have continued to widen globally, from 3:1 in 1982 to 5:1 in 2001 to 13:1 in 2019.

At the country level, F:M differentials are highest in countries with very high tubectomy prevalence and very low but measurable vasectomy prevalence (e.g., 76:1 in India and 36:1 in the Dominican Republic). India’s tubectomy prevalence of 38.0% represents three-fourths of all modern method use and equates to more than 100 million tubectomy users, compared to only 710,000 vasectomy users (0.3% vasectomy prevalence). Similarly, in China, where tubectomy prevalence is 18.3%, 48.5 million WRA use tubectomy and 3.8 million rely on (a partner’s) vasectomy. Four other Asian LMICs have double-digit tubectomy prevalence with much lower vasectomy prevalence. In LAC countries with higher vasectomy prevalence—Brazil, Colombia, Costa Rica, and Mexico—tubectomy use is 4–16 times higher than vasectomy use. Vasectomy prevalence increased while tubectomy prevalence decreased in 4 LAC countries: Colombia, Costa Rica, Ecuador, and Panama. In Africa, 1 country, Malawi, also has substantial tubectomy prevalence (10.9%), with a vasectomy prevalence of 0.1%.

### Correspondence of Vasectomy Prevalence and Gender Equality

Countries that rank among the highest in vasectomy prevalence rank among countries with the highest gender equality and vice versa (Table 2).^15^ Conversely, in 57 of the 62 countries with the lowest gender equality, vasectomy prevalence is low to nonexistent. (While analysis of equity differentials in PM use within countries is beyond the scope of this article, we were surprised to find that in low-PM use Nigeria and Kenya as well as in high-PM use India, there was generally little to no measurable difference in vasectomy prevalence according to wealth quintiles, urban-rural residence, or education levels, although there were clear—and expected—gradients according to age and parity.)^21^^–^^23^

Overall, among 91 countries with adjusted rankings on GII, 3 of the 5 highest-ranked countries and 8 of the 10 highest-ranked countries on GII are among the 10 countries with the highest vasectomy prevalence.^15^ The 2 exceptions, Sweden and France, ranking very high, 1st and 4th, respectively, on gender equality, but have lower rankings, 18th and 20th, respectively, on vasectomy prevalence. The Netherlands and South Korea ranking 2nd and 5th, respectively on adjusted GII, and ranking 10th and 1st on vasectomy prevalence, respectively. The next 5 highest-ranked countries on gender equality—Spain, Canada, Australia, the United Kingdom, and New Zealand—ranking 6th to 10th on vasectomy prevalence, respectively. Among the 10 countries with the highest rankings on vasectomy prevalence, the only 2 countries with rankings below 10th in adjusted GII are the United States, 4th in vasectomy prevalence and 12th in adjusted GII, and Bhutan, 3rd in vasectomy ranking but only 38th in adjusted GII ranking. In 8 of the 10 countries with highest gender equality (on adjusted rank), vasectomy prevalence exceeds tubectomy prevalence, and both PMs are used substantially.

The readily apparent correspondence between countries ranking highly on both gender (in)equality and vasectomy prevalence would likely be even more pronounced if Switzerland, Denmark, and Norway—first, second, and fifth in UNDESA’s ranking of GII (among 162 countries), respectively—had not needed to have been excluded from consideration of adjusted country rankings on methodological grounds (either for not having a recent survey or not providing method-specific values for PMs).^15^ For at least 3 decades, these 3 countries have had societal norms of high modern contraceptive use (65%–78% MCPR), small desired family size, and high PM use: 17%–21% prevalence, including very substantial vasectomy prevalence of 6.3% in Norway (1998), 8.3% in Switzerland (1995), and 10.0% in Denmark (1993), respectively. If those vasectomy prevalence levels pertained to more recent surveys, these 3 countries would rank among the world’s 10 highest in vasectomy prevalence. By the mid-1990s, vasectomy use was 61% of tubectomy use in Norway and Switzerland and more than twice tubectomy use in Denmark.

In general, rankings on GII for LAC and Asian countries, almost all of which have high PM prevalence and measurable vasectomy prevalence, fall between those of HICs and less developed countries, most of which have low to negligible vasectomy prevalence of 0.1% or less. LMICs with high vasectomy prevalence rankings but lower (midrange) rankings in GII include Bhutan, Brazil, Costa Rica, Mexico, and Nepal. Costa Rica, Brazil, and Colombia, ranking 12th, 13th, and 14th in the world, respectively, with the highest vasectomy prevalence, ranking 17th, 31st, and 34th, respectively, in adjusted GII. Among LAC countries, Costa Rica has the highest vasectomy prevalence and the second-highest adjusted (and non-adjusted) GII ranking.

## DISCUSSION

The international FP/RH community, in our experience, recognizes that vasectomy has low prominence, availability, demand, and use in FP programs in LMICs. Furthermore, a decline in vasectomy’s method share, “from low to lower,” was 1 of 4 key trends noted in a 2020 analysis of changes in contraceptive method mix in recent years.^24^ However, the starkness and extent of vasectomy’s marginal situation, worldwide and in almost all LMICs, has perhaps not been fully appreciated, as it was not by the authors before embarking on this article.

As seen in our findings, vasectomy is faring poorly today in most LMICs in terms of its being a method norm that people know about, correctly understand, consider, might choose, and then would be able to access and use. While there is no “ideal method mix” or prescribed vasectomy prevalence that “should” be attained, vasectomy’s negligible to nonexistent presence in the method mix and FP programming of most LMICs is clearly suboptimal if method choice is to be broadened and constructive male engagement in FP encouraged.

To be clear, widespread tubectomy availability and access is a good thing: tubectomy’s substantial—and voluntary, informed—use, like all FP use, saves women’s lives.^25^ In 2017, the MMR in some LMICs exceeded 1 maternal death for every 100 births,^26^ and in all LMICs, maternal morbidity exceeds maternal mortality. Rather, vasectomy use represents a true partnership in a couple’s shouldering of contraceptive responsibilities; however, male-to-female disparities in PM use have widened, not narrowed, over the past 3 decades.

### Why Has Vasectomy Been So Difficult to Add to an LMIC’s Method Mix?

As described in this journal^27^^,^^28^ and elsewhere,^29^ there are many demand-side, supply-side, and normative challenges to vasectomy truly becoming an available and accessible method option among the range of options people consider and use. These challenges, which account for vasectomy’s lack of availability in LMICs, are easy to identify but difficult to address. They occur in the following contextual and programming domains:

#### Societal Norms and Contraceptive Responsibilities

In most LMICs, FP, like child-rearing, has largely been seen as a woman’s responsibility and domain.^30^ Certainly, the demands of pregnancy and the brunt of pregnancy-related morbidity and mortality fall on her. Consequently, FP service provision has long been oriented to reproductive-age women, and women are the main users of contraception, including PMs. Health care programs generally focus to a lesser extent on men’s health care needs,^31^ and services for men in FP settings are typically limited. That there are only 2 contraceptive methods for men, both with method-related challenges—the condom, which is nonclinical and has a failure rate in typical use of 5%–13%,^2^ and vasectomy, which must be surgically delivered, is suitable only for limiting fertility, and requires a skilled provider and supportive clinical infrastructure—is also likely a relevant factor in these dynamics.

#### Awareness and Accurate Understanding

Vasectomy has long been and still is the least known modern method in LMICs.^3^^,^^4^ For example, in Africa’s most populous country, Nigeria, only 18% of women and 33% of men know of vasectomy.^21^ In contrast, the most widely known method there, the pill, is known by 82%–88% of women and men, injectables are known by 82%–88% of women, and tubectomy is known by 48%–49% of married people. Similarly, in Kenya, only 47% of women and 56% of men know of vasectomy, whereas 84%–87% know of tubectomy and 97% know of the pill.^22^ Even in countries where PMs are much more widely used, awareness of vasectomy is lower than that of tubectomy. For example, in India, 93%–98% of urban men and women know of tubectomy, whereas only 85%–88% know of vasectomy.^23^ In Colombia, where awareness of tubectomy is essentially universal (98% among MWRA), awareness of vasectomy is similarly lower (89%).^32^ Differences in how providers inform clients about PMs may contribute to such low awareness: one study from the United States found that women received more counseling from their providers on PMs than did men.^33^ Studies in Ethiopia found knowledge of vasectomy to be associated with an individual’s level of education,^34^^,^^35^ positive attitude toward vasectomy, and intention to use vasectomy.^36^^,^^37^

Furthermore, in surveys of contraceptive use, “knowledge” of vasectomy relates to whether respondents have heard of or are aware of the method but not necessarily to whether they have accurate or correct understanding of it.^38^ Vasectomy is the method most plagued by widespread, deep-seated, and persistent myths and misunderstandings about it and biases against it.^4^^,^^27^ People erroneously fear that vasectomy might make a man impotent or “weak,” promiscuous, and/or unable to work and contribute to family well-being. Not only do FP clients or potential clients have deep-seated biases against vasectomy, but health care providers and program leaders often have biases against it as well.^28^^,^^39^ We have even encountered translators who refused to translate the word “vasectomy” in a discussion of method options because they thought what was being discussed was castration.

Not only do FP clients or potential clients have deep-seated biases against vasectomy, but health care providers and program leaders often have biases against it as well.

#### Client Demand and Program Prioritization

The normative realities and dynamics discussed earlier lead to a general lack of demand for vasectomy, which has been identified as a major impediment to greater interest in vasectomy among current and potential future FP clients in LMICs.^27^ Reflecting (and also contributing to) this lack of demand among women and men alike, prioritization of vasectomy has generally been low in FP programming and donor funding.^40^ Low prioritization, over many decades, is also implicit in the low vasectomy prevalence rates seen in almost all LMICs in multiple contraceptive use surveys from the 1970s through the early 2020s.^11^ A recent informal analysis found that between 2005 and 2015, one major bilateral FP donor had only 5 projects with a vasectomy component, among upwards of 30 FP projects; this and other analyses confirm that funding for vasectomy has been meager.^39^^,^^40^

On the one hand, low program and donor prioritization are understandable and defensible. Funding for FP has generally been limited, FP programs aim to serve as many people as possible with limited resources, and vasectomy uptake, at least in initial years of programming, will almost certainly be modest. On the other hand, absent support for and attention to vasectomy, its marginal status will not improve. In 2020, the U.S. Agency for International Development (USAID)–funded MOMENTUM Safe Surgery in Family Planning and Obstetrics project, which supported the development of this article, was awarded to “build awareness of, equitable access to, and provision of high-quality surgical care,” including (both) PMs as a priority component.^41^ Funding for vasectomy at the service level, however, will depend on decisions taken at individual USAID Missions and be context driven, typically entailing an intent to achieve prompt and substantial results in service uptake.)

#### Advocacy and Demand-Side Efforts

**FIGURE 2:**
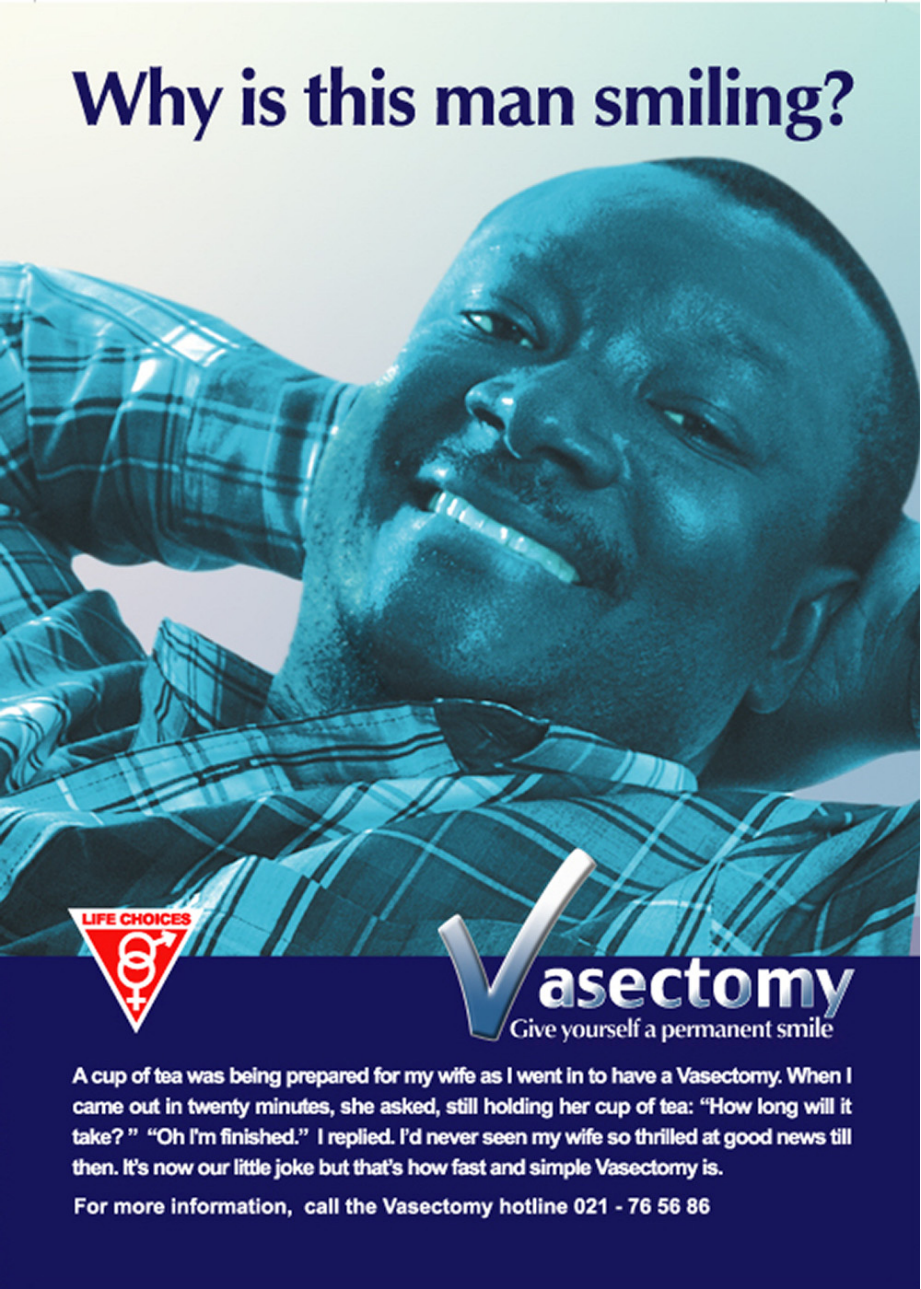
Poster From Vasectomy Demand Creation Campaign in Ghana Source: The ACQUIRE Project/EngenderHealth via Knowledge SUCCESS (reproduced with permission).^42^

**FIGURE 3:**
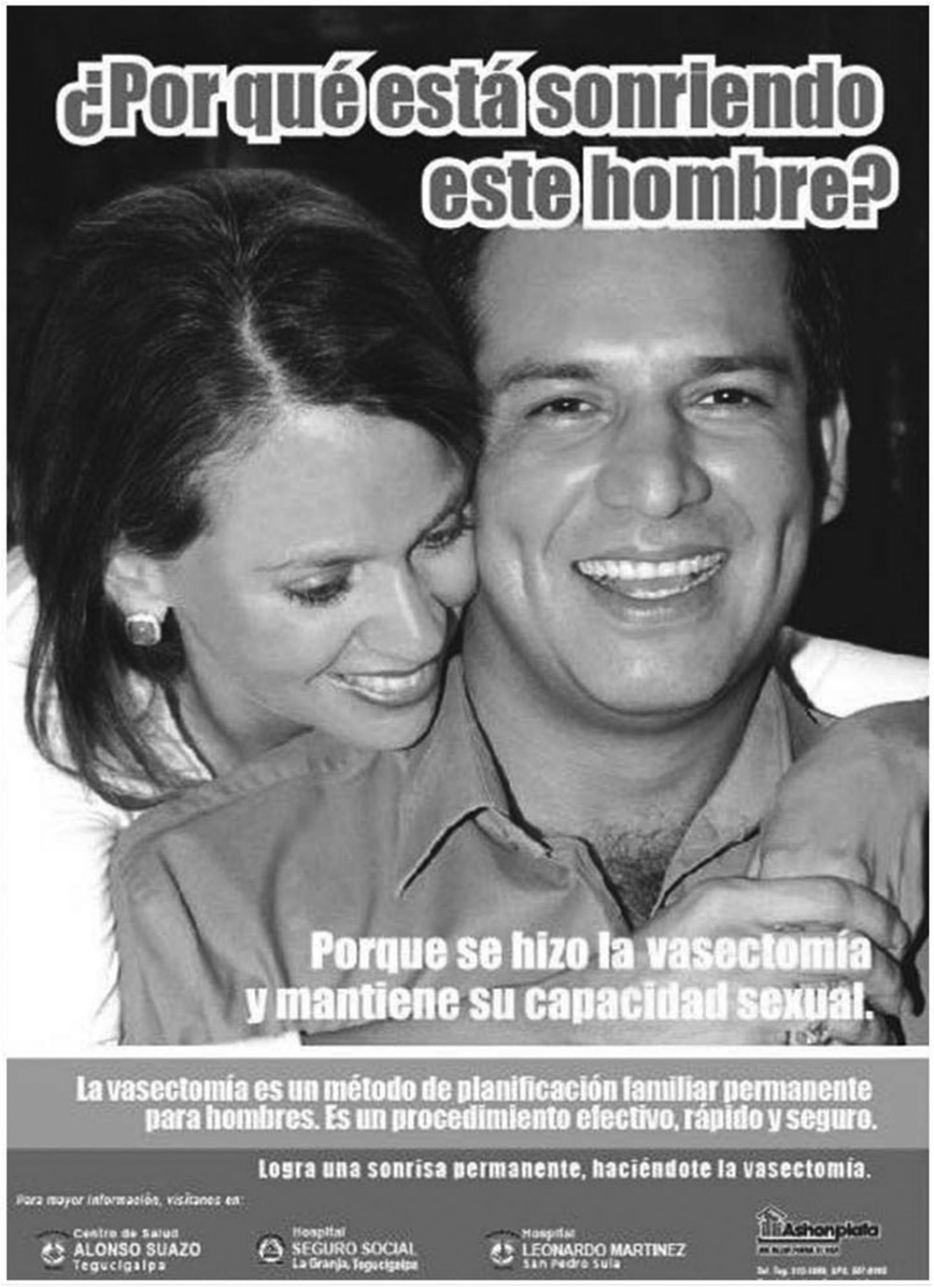
Poster From Vasectomy Demand Creation Campaign in Honduras Source: The ACQUIRE Project/EngenderHealth via Knowledge SUCCESS (reproduced with permission).^43^

**FIGURE 4:**
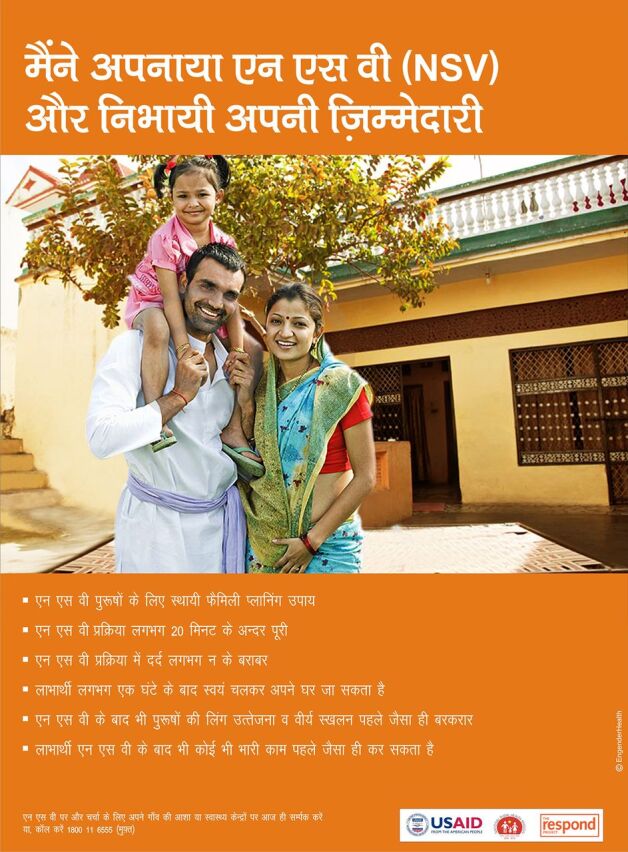
Poster From Vasectomy Demand Creation Campaign in India Source: The RESPOND Project/EngenderHealth (reproduced with permission).^45^

Less programmatic attention has been given to advocacy and demand-side efforts,^9^^,^^40^ whose effects are upstream from supply-side interventions that may generate more immediate outcomes in terms of trained providers and new vasectomy users. Consistent advocacy and demand-side efforts could be of greater ultimate impact; however, donor funding for these elements has been sporadic and limited. When funding has been made available, demand-side efforts via multiple communication and messaging have been very effective and successful on their own terms (see discussion of Brazil). Posters developed by 2 USAID-funded projects (ACQUIRE [Access, Quality, and Use in Reproductive Health] and RESPOND [Responding to the Need for Family Planning]) between 2008 and 2014 in Ghana, Honduras, and India that addressed the demand-related aspects of vasectomy as part of holistic vasectomy programming are still relevant and could be useful today.^42^^,^^43^ The pictured individuals, including a Ghanaian mayor (Figure 2),^44^ were themselves satisfied vasectomy adopters and vasectomy champions. These images directly speak to people’s concerns regarding vasectomy by conveying images of “strength”; joyful, harmonious couples (Figure 3)^43^; and happy, coherent (and small) families (Figure 4).^45^ Vasectomy provision in project-served areas doubled or tripled during these efforts. Demand-side aspects of vasectomy programming have also been a prominent feature of the work of World Vasectomy Day and Marie Stopes International (both discussed later), coordinated with provider training activities and concurrent and subsequent service provision.

#### FP Service Delivery

Because client demand for vasectomy has been low to negligible at the population level in most LMICs, vasectomy services have been sparse, and, as noted earlier, bias against it is often found among providers as well as clients.^3^^,^^4^^,^^27^^,^^29^ Even when men and couples do have an interest in vasectomy, the availability of knowledgeable, trained, and skilled providers to discuss vasectomy with potential clients or provide it to those who would choose it is generally low at service sites. At the program level, the modest donor funding made available to international nongovernmental organizations (NGOs) working on vasectomy in LMICs has mainly been devoted to provider training and research on details of the surgical procedure itself.^28^ These supply-side aspects can be addressed relatively quickly and tangibly, although low demand (and therefore few clients) impedes clinical training efforts. With few clients, it can also be difficult for providers to maintain newly acquired surgical or counseling skills.

#### Metrics of Program Achievement and “Success”

Program leaders and donors generally want measurable and substantial results that will quickly address people’s ongoing and unmet needs for contraception. Common metrics for success in FP programming include “increases in contraceptive uptake,” “cost effectiveness,” “low cost per user,” and similar metrics reflective of prompt results. Prospects for scale-up and sustainability are also important considerations. However, these metrics, as well as the general criteria underlying programming with limited funds—of aiming to help the most people—reasonable as they are, work to vasectomy’s disadvantage. At the program level, it can take several years of tireless work spearheaded by vasectomy champions before vasectomy becomes more widely, substantially, and routinely available in a country’s method mix. At the individual level, the process whereby men and couples move from considering to obtaining a vasectomy also can take several years.^3^ Furthermore, men often learn of vasectomy from other men who have previously had the procedure; this is difficult when vasectomy prevalence is very low and few men have already accessed it. Metrics related to advocacy events, the (sustained) work of vasectomy champions, and information-providing or demand-generating activities (via multiple communication channels, e.g., blogs, newspaper articles, call-ins, and in-person interactions.) would also be useful and relevant measures bearing on success in helping vasectomy to become truly part of a country’s available method mix, thereby broadening people’s contraceptive options.

Men often learn of vasectomy from other men who have previously had the procedure; this is difficult when vasectomy prevalence is very low and few men have already accessed it.

The contrast between 2 successful program efforts in Rwanda and Ethiopia illustrates vasectomy’s relative disadvantage in terms of the extent and rapidity of uptake of a programmatically new, highly effective method that affords several years or more of contraceptive protection and broadens the available method mix. In Rwanda, a substantial training effort—successful in context and demonstrating that African men, like men elsewhere, would choose vasectomy—led to more than 2,500 vasectomies being performed between 2010 and 2012.^46^ At 0.2%, Rwanda’s vasectomy prevalence is one of the highest in Africa, and we estimate about 7,000 Rwandan women are now relying on a partner’s vasectomy (of 3.3 million WRA).^12^ In Ethiopia, however, for a variety of interrelated reasons having to do with client preferences, gender norms around who uses contraception, mode and ease of method delivery, and program and donor prioritization, more than 1 million women accessed a hormonal implant between 2009 and 2015 when that method became much more widely available due to lowered commodity cost and ease of training multiple service providers.^47^ Rapid uptake of implants has also occurred in other countries,^48^ and this long-acting reversible method is likely serving some people’s reproductive intentions to limit further births, which may dampen demand for PMs

#### Cost Considerations

Vasectomy has been analyzed to be among the most cost-effective of all contraceptive methods per couple-year of protection when added (and prorated) to an existing program.^49^ However, the cost of a stand-alone vasectomy program would likely be greater, entailing the full cost for facilities, service providers, and related demand-side activities. The rate of vasectomy uptake also needs to be factored in.

#### Concerns About a “Single-Method Focus”

We have heard program leaders and donors who are sympathetic to the need to broaden vasectomy availability and access still indicate reluctance to support “single-method efforts” or “a single-method project” that would focus on vasectomy advocacy or programming. Because a fundamental guiding principle of international FP programming is to ensure broad method choice, they are concerned that such an approach might privilege a single method. From the standpoint of vasectomy, however, this stance can be problematic: absent a focused and steady, sustained effort for this single method, vasectomy’s prospects at the program level—to become a truly available and accessible method option on the menu of client choices in FP programs—will likely remain as poor going forward as they have proven to be during the past 4 decades. A broader male RH project that might surmount this “single-method objection” could address vasectomy, although the typical male client interested in vasectomy has a different profile than, say, a candidate for male circumcision or a seeker of services for sexually transmitted infections or HIV. We also note that some single-method projects (e.g., for the Standard Days Method) intended to add a less well-known method to the mix of available client options in LMICs have long been donor-funded.^50^

#### Concerns About Coercion

Forced sterilization has a long and sordid history that may be influencing clients, potential clients, programs, and donors. A bedrock principle of FP programming and service provision is that clients must have free, informed, and voluntary choice in contraceptive decision-making and use. This is particularly and most fundamentally the case with PMs, which must be provided without any undue influence or frank coercion.^3^ Forced sterilization has occurred during the past 50 years or longer in the United States, Canada, China, India, and elsewhere. The eugenics movement, which arose and flourished during the 19th and 20th centuries in the United States, often entailed forced sterilization, mainly of women; it only ended officially in 1981 and has been reported subsequently.^51^ Similarly, forced sterilization occurred in Canada, with indigenous women disproportionately targeted.^52^^,^^53^ Coercive efforts to force sterilization on people that occurred in India in the 1970s, in a widely publicized campaign aimed mainly at men, still have an impact on the Indian public today.^54^^,^^55^ This legacy may be exerting an “inverse halo” effect on demand for vasectomy in India, where use of PMs at relatively low parity and age is a societal norm and tubectomy use is extensive, yet vasectomy use is very low and declining. In China, where tubectomy use is also extensive, recent allegations of forced sterilization of minority women have been prominent.^56^

Forced sterilization has a long and sordid history that may be influencing clients, potential clients, programs, and donors.

Even the word “sterilization” is problematic, conjuring memories of compulsory sterilization programs directed widely at religious and racial minorities.^57^ Because of these resonances and to avoid inadvertent stigma, in this article, we have used the term “tubectomy”—which is parallel to “vasectomy.” (The surveys compiled by UNDESA that we rely on extensively report prevalence figures for “female sterilization” and “male sterilization.”) Similar sensitivity to language accounts for avoiding the word “targets” in PM programming, as opposed to “goals,” “objectives,” or “desired results”—and then ensuring that program activities protect and reinforce voluntary choice. It is important for FP programs in LMICs to be mindful of these linguistic considerations and the still-potent negative resonances of unacceptable practices, while still working to make vasectomy a true method option for the people they serve.

### Success Stories: Some Countries Have Done Well

Despite the largely disheartening findings documented in this article about vasectomy’s low prevalence of use and the many challenges to its programming in most LMICs, all is not bleak. Some current and former LMICs have already made noteworthy progress in incorporating vasectomy as an available and acceptable method option that many people are accessing. In addition, several organizations devoted generally to service provision of underavailable clinical FP methods or specifically to increasing vasectomy awareness and availability are successfully helping to increase vasectomy uptake. We highlight some of these countries and organizations, emphasizing aspects and approaches likely to be of most relevance to expanded vasectomy programming in LMICs.

#### South Korea

South Korea’s contraceptive method mix is a rare exception to the pattern of higher reliance on female methods that predominates in almost all countries. The male condom and vasectomy are the 2 most commonly used methods, with method shares of 36.6% and 25.3%, respectively. South Korea ranks first in vasectomy prevalence among the world’s 190+ countries and tenth in gender equality among 162 countries—the highest ranking in gender equality of any Asian country.^15^ South Korea’s last survey providing method-specific disaggregation is from 2009, but in 3 subsequent surveys, comparable or higher overall contraceptive use was registered (82.3% contraceptive prevalence rate in 2018). Vasectomy prevalence has been in double digits in 7 surveys since 1988, and male condom use has also been substantial, strongly suggesting that constructive male engagement and sharing of contraceptive responsibilities is an entrenched societal and contraceptive norm. South Korea’s socioeconomic ascent from LMIC to HIC and its high gross domestic product (GDP) per capita (US$31600 in 2021) coincide with these achievements and reflect its attainment of a “demographic dividend.”^58^^,^^59^

South Korea’s contraceptive method mix is a rare exception to the pattern of higher reliance on female methods that predominates in almost all countries: male condom and vasectomy are the 2 most commonly used methods, with method shares of 36.6% and 25.3%, respectively.

#### Bhutan

In Bhutan as in South Korea, a couple’s choice of vasectomy after attaining desired family size is a longstanding national norm, vasectomy remains the predominant PM used. One of every 5 MWRA contraceptive users in Bhutan relies on vasectomy, and Bhutan has the third highest vasectomy prevalence in the world, higher than that of more than 190 countries. Bhutan has maintained a double-digit vasectomy prevalence of 12%–13% over a recent decade while more than doubling its MCPR to 65.4%, a level of modern method contraceptive use greater than that of Australia and Spain and comparable to that of the United States (66.1%). A 2008 summary analysis found a number of factors in Bhutan that other countries could consider when seeking to improve vasectomy availability in their contraceptive method mix^60^:

- Women encourage their partners to be sterilized because vasectomy is easier [than tubectomy].
- Community attitudes are accepting of men who have had vasectomies.
- Vasectomy services are of good quality, so there is a pool of satisfied clients available to motivate other men to have the procedure.
- Men routinely accompany women during childbirth, a sign of their willingness to share family responsibilities.
- The government provides free vasectomy services and offers seasonal vasectomy camps—temporary service sites that are convenient for villagers to access.

Perhaps not unrelated, Bhutan’s constitution commits its government to promoting conditions that enable the pursuit of Gross Domestic Happiness.^61^ These notable achievements regarding vasectomy occurred despite Bhutan being a poor country, with a GDP per capita of US$2193 at the time of its most recent survey of contraceptive use in 2010^62^ and ranking 127th in 2017 on the UNDP Human Development Index.^15^

#### Brazil

**FIGURE 5:**
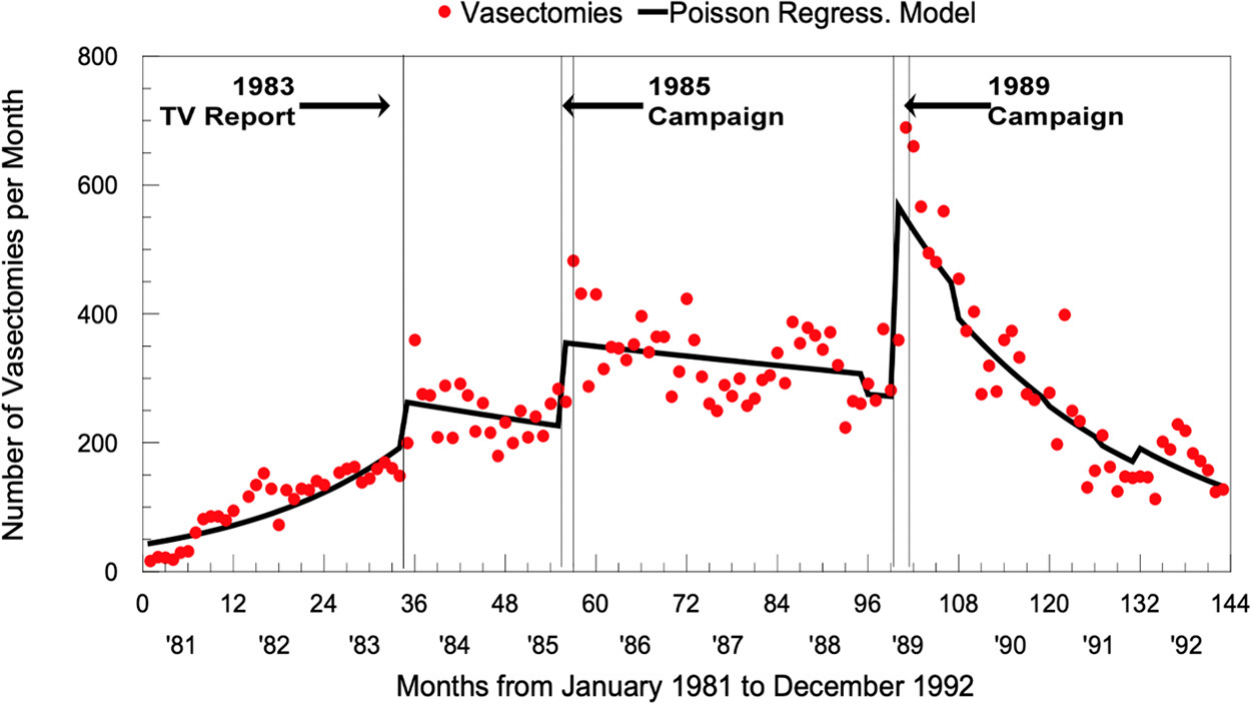
Increase in Vasectomies Following Media Events Promoting Vasectomy in São Paolo, Brazil Adapted from Kincaid et al., Impact of a Mass Media Vasectomy Promotion Campaign in Brazil, (JHU/SPH/CCP & PRO-PATER) with permission.^63^

The results of a successful mass media effort mounted in the late 1980s in Brazil’s largest city, São Paulo, convey 3 important lessons still relevant today. First, they demonstrate the power of mass media to affect vasectomy-related behavior, not only knowledge.^63^ As seen in Figure 5, uptake in vasectomy ensued, first after a TV report and then more markedly during each 6-week period that a mass media campaign was conducted. Television also supplanted interpersonal communication as the chief means of conveying accurate knowledge about vasectomy as a method and available service. Second, vasectomy uptake receded to precampaign levels each time the campaigns ended, underscoring why media-driven efforts need to be maintained (Figure 5). Third, this effort, which “promoted male responsibility in a culture celebrating machismo,” was largely implemented by vasectomy-provider champions, themselves Brazilian, including a charismatic physician who led a Brazilian organization, Pro-Pater, that provided male RH services more generally as well as vasectomy. Concomitant with this demand-side effort, vasectomy prevalence tripled between 1986 and 1996, from 0.8% to 2.6%, and doubled again by 2007 to 5.1%. The 2.5 percentage-point increase in 2007 coincided with an 11 percentage-point fall in tubectomy prevalence. Brazil has maintained a vasectomy prevalence of 4%–5% during the past decade and a half, suggesting normalization and sustainability of vasectomy as a method choice regularly considered by Brazilian couples.

#### Colombia

In LAC, where there is a high prevalence of PM use but less gender equality, Colombia doubled its vasectomy prevalence between 2005 and 2016, from under 2% to almost 4%. The preponderance of FP service provision has been provided by an NGO, Profamilia, an International Planned Parenthood Federation affiliate. Long a pioneer in championing FP in Colombia and LAC, Profamilia has been a predominant provider of contraceptive services, including provision of PMs, making services available and affordable.^64^ Vasectomy’s 1.8 percentage-point increase in prevalence constituted 23% of Colombia’s overall increase in MCPR; vasectomy’s method share rose by 81%; and the F:M differential in PM use declined by almost half, from 17:1 to 10:1—all promising trends and illustrative of the contributions that can be made by the private sector in an LMIC.

Colombia doubled its vasectomy prevalence to almost 4%, with most of the vasectomy services provided by an NGO.

#### Costa Rica

Costa Rica has maintained the highest vasectomy prevalence and method share in LAC since 2010 (in 4 consecutive surveys) and has the highest adjusted rank in GII of any LAC country. The F:M ratio in PM use has narrowed from 6:1 in 2010 to 4.2:1 in 2018 (mainly due to a falloff in tubectomy prevalence). A 2016 issue brief analyzing several decades of USAID assistance to Costa Rica’s FP program noted several reasons that FP thrived there.^65^

- Programs reached all societal levels with high-quality, voluntary FP services aided by a strong social policy framework [that included FP].
- The partnership between the Government of Costa Rica, USAID, and NGOs led to an increase in modern contraceptive use between 1970 and 2015.
- A second project advocated universal coverage of FP.
- Subsequent projects focused on reaching specific client groups and strengthening health systems by improving the quality and availability of services and providing information and counseling to clients, which increased contraceptive uptake by both urban-educated women and rural, more disadvantaged women.

These approaches to ensure wide provision of accurate information and counseling on method options, method use, and side effects management; strengthen the various components of a health system to ensure quality and availability of services; and secure universal coverage for vasectomy (as well as for all other FP options) in insurance and other payment mechanisms, would be important in any country to increase vasectomy availability and access. (In the United States, while female sterilization [tubectomy] and other contraceptive methods for women are mandated to be covered under the Affordable Care Act, a 2021 clarification confirmed that male sterilization [vasectomy] is not included in the mandate.^66^)

#### World Vasectomy Day

During the past decade, a multinational network of vasectomy champions has mounted World Vasectomy Day (WVD), with activities to further vasectomy awareness, access, and service provision occurring throughout the year in the United States, Canada, and a number of LMICs.^67^ Funded by small grants and volunteering their services, WVD master trainers have trained interested service providers who subsequently have trained others, leading to more than 70,000 vasectomy procedures being provided between 2013 and 2020 in Bolivia, Colombia, Ecuador, Mexico, Indonesia, Rwanda, and elsewhere. Demand-side activities, led by in-country local groups and institutional partners, are also an important part of WVD’s holistic approach. They have used multiple social and mass media channels (e.g., Twitter, Facebook groups, blogs, radio and TV interviews) innovatively and to wide effect, prompting thousands of contacts from individuals interested in vasectomy, as well as recent pieces in influential newspapers and magazines.^68^^,^^69^ Men and women are informed about what vasectomy is and is not, and potential clients are able to locate and access vasectomy providers. Satisfied vasectomy clients are also engaged as vasectomy promoters and champions. At recent meetings of the International Conference on Family Planning, several vasectomy clients and their providers in the United States were interviewed via teleconference in real time during the client’s vasectomy procedure. In addition to WVD’s fruitful collaboration with Marie Stopes International (MSI)/Bolivia (discussed later), a recent and notable partner achievement has been that of the Mexican Ministry of Health. In 2021, the Ministry of Health provided vasectomy to a monthly average of about 3,500 men from every Mexican state, including 6,600 men accessing vasectomy in November 2021 during WVD’s active week of programming there (written communication, Jonathan Stack, Cofounder, WVD, November 2022, with screenshot of data from the Sistema Nacional de Información Básica en Materia de Salud of Mexico’s Centro Nacional de Equidad de Genero y Salud Reproductiva).

#### Marie Stopes International

MSI, an international NGO, has been a global leader in vasectomy service provision. Between 2016 and 2021, MSI supported vasectomy services in 26 countries, 22 of them LMICs (written communication, Anna Mackay, Director, Private Foundations, MSI, December 6, 2022). During that 5-year period, more than 225,000 men received a vasectomy via static clinic networks, mobile outreach operations, or capacity-building of public sector providers. Services were supported on the demand side by a range of awareness and promotion activities relying on social media, mass media, and/or community outreach. MSI’s main country partner among LMICs has been Bangladesh,^70^ with more than 145,000 vasectomies having been supported from 2016 to 2021. Substantial reductions in donor funding, however, curtailed MSI’s mobile outreach services and led to a marked decline, from more than 60,000 procedures performed in 2016 and 47,000 in 2017 to fewer than 4,500 performed in 2020 and 2021. This decline, plus the retirement of experienced public sector vasectomy providers and an increased program focus on hormonal implants, were the main contributors to Bangladesh’s decline in vasectomy prevalence, from 1.2% in 2011 to 0.5% in 2019. MSI also served almost 10,000 men with vasectomy in Nepal, which has the highest (though declining) vasectomy prevalence in Asia.

A more positive outcome occurred in Papua New Guinea, where almost 8,000 men chose vasectomy between 2016 and 2021.^71^ This contributed considerably to Papua New Guinea’s having attained the third highest vasectomy prevalence in Asia (0.8%; 2018). This success, notable in a country with one of the world’s highest levels of gender inequality, also entailed training men who were satisfied users of vasectomy to facilitate discussions with other men from their community, including challenging and attempting to change negative and harmful gender norms associated with masculinity.

### How Many Procedures and Providers Might Be Needed to Reach 1% Vasectomy Prevalence?

The recent experience of MSI/Bolivia in expanding access to vasectomy services holds promise as an emerging success story. It also provides suggestive evidence that relatively few trained vasectomy providers and vasectomy procedures may be needed for a country to attain a modest 1% level of vasectomy prevalence. After having been trained in vasectomy by WVD’s master trainers in November 2021,^72^ 4 MSI/Bolivia FP providers trained 10 additional FP providers over the ensuing months, enabling the addition of vasectomy services to 4 MSI/Bolivia mobile teams and 6 fixed facility sites (written communication, Ana Cecilia Velasquez Rossi, Country Director, MSI/Bolivia, December 8, 2022). The 10 providers were trained at various times, which resulted in 6.7 person-years of service after training. This was complemented by an active vasectomy awareness campaign through social media and the press. As a result of the training, and no longer needing to rely on urologists, MSI/Bolivia was able to lower the price of vasectomy by around 50% in fixed clinic sites while also maintaining a subsidy program and sliding scale of charges there. (All methods, including vasectomy, are provided at no cost to clients in mobile units.) In the 12-month period from December 1, 2021 through November 30, 2022, 906 vasectomies were provided by these 10 providers, a 12-fold increase in vasectomy provision compared to the 2019 level of vasectomy provision by MSI/Bolivia (77 vasectomies), suggesting that latent demand for vasectomy is being met.

Hypothetically, doubling the number of providers from 10 to 20 would lead to 2,938 vasectomy clients served annually (assuming they provide vasectomy during the entire year and with the same level of clinical support and awareness promotion). Applying the calculations described in the Methods section and maintaining this number of clients over time—on average, fewer than 1 vasectomy per provider per working day—would result in a national vasectomy prevalence of 0.5% after 5 years, 0.9% after 10 years, and 1.2% after 15 years. Countries with populations larger than Bolivia’s (12 million in December 2022) would require proportionally more service providers to achieve a 1% prevalence of vasectomy use during the same time span. Likewise, countries with smaller populations would require proportionally fewer vasectomy providers to do so. Also, countries may have people and programs with higher or lower levels of receptivity to vasectomy services compared to Bolivia’s, which would affect these estimates. Still, the MSI/Bolivia experience suggests the potential for progress in making vasectomy a true method option in LMICs, even in countries with low gender equality and GDP per capita. (Bolivia’s values for these indicators are among LAC’s lowest.)

## PROGRAM AND POLICY IMPLICATIONS

**FIGURE 6:**
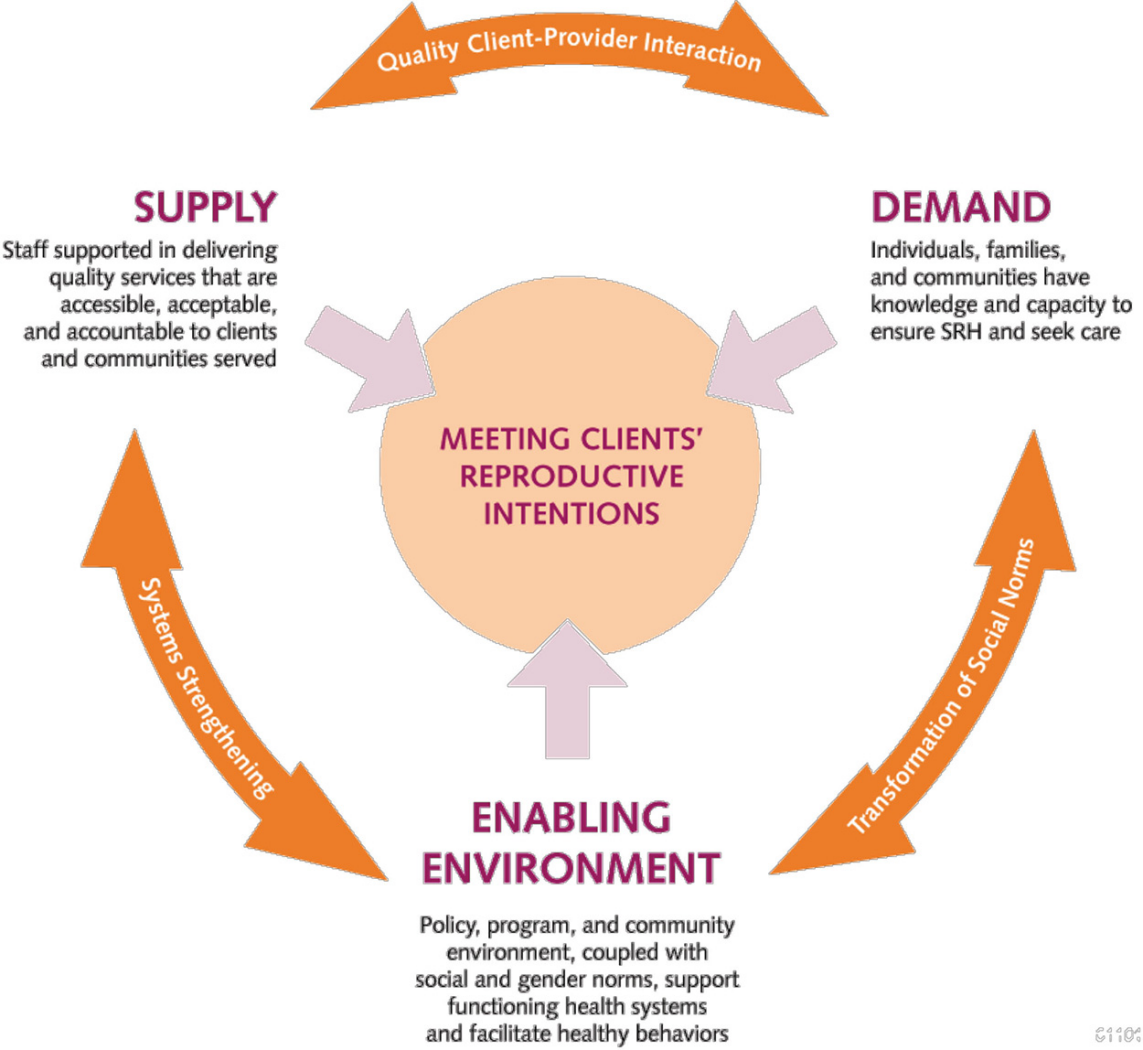
Holistic Supply-Enabling Environment-Demand Model for Family Planning Abbreviation: SRH, sexual and reproductive health. Source: EngenderHealth.^73^

We offer the following set of policy and program recommendations for those hoping to help vasectomy truly be on the menu of client contraceptive options in LMICs. These recommendations are ordered (roughly) according to EngenderHealth’s SEED Model (Supply-Enabling Environment-Demand), a holistic conceptual framework for FP programming that we have found useful (Figure 6).^73^

- Programming efforts need to be coordinated between demand-side, supply-side, and advocacy interventions, as the situation warrants.
- Advocacy for vasectomy at all levels of the FP ecosystem is important, especially to attain funding that is additive, not substitutive, to existing FP program budgets.
- Demand-side work needs to be a substantial part of vasectomy-related programming, including focusing on women as well as men, and addressing gender constraints.
- Harnessing mass and social media channels and approaches (e.g., TV interviews, radio spots, SMS messaging, Facebook groups, and blogs) to increase accurate knowledge of vasectomy as a method and link prospective clients to vasectomy service providers is particularly effective and cost-effective per person reached.^28^
- Training efforts, when indicated, should entail a whole-site training approach and use cascade training (with those trained then training others). This should be coordinated with community/client demand-side work.
- Vasectomy champions are needed at all levels—policy, program, client, community, and donor. This includes service providers and satisfied male users willing to discuss their vasectomy with others in their community.
- Among providers, a linked “network of vasectomy champions” could be helpful, leveraging the global health and FP/RH communities’ growing interest in safe surgery.^74^
- Vasectomy services, which should be affordable if not (close to) free of charge, should be provided via diverse modalities depending on the country context (e.g., static clinics, mobile outreach, and, after capacity-building, in the public sector). In harder-to-reach settings, mobile services have been found to be particularly effective.^75^
- Follow-up support to vasectomy clients could utilize various media applications (e.g., SMS, Facebook groups, and telemedicine) to reinforce clients’ correct knowledge of vasectomy and reassure or assist them if any minor side effects from the procedure arise.
- An innovative service delivery approach in a country where PM use is a norm might be to promote “postpartum vasectomy”: the 3-month immediate postpartum period (a “fourth trimester”)—when newly delivered mothers are focused on their own recovery and couples tend to be most interested in limiting subsequent births—dovetails with the time an additional contraceptive method is needed before a vasectomy becomes fully effective.
- “Success” should be reframed, with achievement of immediate increases in vasectomy uptake secondary to laying a foundation for a longer glide path leading to normalization and sustainability of vasectomy within a country’s method mix. Appropriate indicators could be ones related to information transfer and receipt, demand creation, broadened method choice, increased program capacity to provide vasectomy regularly and routinely, and greater gender equality in PM use among couples wishing to limit childbearing.
- Two small but easily implementable and useful survey-related recommendations are to (1) ensure all surveys of contraceptive use provide values for vasectomy, even if its prevalence is negligible—this conveys the expectation that vasectomy should be an available method option in the country; and (2) replace the term female sterilization with tubectomy to avoid stigma and to parallel the term vasectomy.
- A vasectomy-focused initiative, done appropriately and consistently, is needed to help vasectomy become better known and understood, more wanted, and more accessible to clients in LMICs. This could be done as a stand-alone effort well linked to broader FP programming (or perhaps as part of a broader male RH initiative).
- Programming efforts for vasectomy likely will be “pilots,” so lessons of good pilot programming or “demonstration projects” should be heeded (e.g., make the effort visible in a country’s capital or other main city and via various media); engage respected organizational, medical, and community leaders; and plan for scale-up from the start.

Surveys should always indicate a value for vasectomy even if prevalence is negligible, and use the term tubectomy, not female sterilization.

## CONCLUSION

We are surprised, as we imagine readers may be, at how little reliance there is on vasectomy, not only in LMICs but globally. This is the reality despite increasing contraceptive use, population growth, rising demand to limit further births, and greater equality between women and men—all of which might be expected to increase vasectomy use in LMICs. Indeed, despite the international FP community’s focus on expanding method choice and fostering constructive male engagement and more than 4 decades of funding and programming for FP in LMICs—with noteworthy overall success as a development effort—vasectomy can fairly be characterized as being largely unavailable within the contraceptive method mix offered in almost all LMICs. Furthermore, declines in global vasectomy use are likely to continue over the next few years, given China’s de-emphasis of vasectomy and its accounting for more than one-fifth of global vasectomy use.^19^

Nonetheless, aware of the many challenges faced by vasectomy, we take optimism from those countries—South Korea, Taiwan, and Bhutan in Asia, and Costa Rica, Colombia, Brazil, and Mexico in LAC—that have done well in having vasectomy become and remain a regularly available method option, with men there assuming a greater share of contraceptive responsibility. We would hope that South Asian countries (e.g., India) with their societal norms of PM use and extensive reliance on tubectomy, could become leaders in rights-based provision of vasectomy. Bangladesh might also be able to return to higher provision of access to vasectomy. Similarly, we are encouraged by the early trend to more equitable adoption of PMs among men and women in several LAC countries, with vasectomy garnering a larger share of still-substantial PM use. In Africa, where international donor assistance for FP is now largely focused, efforts to introduce vasectomy more widely are also worthwhile. The program performance of Rwanda regarding vasectomy and Malawi regarding tubectomy, as well as the relatively higher vasectomy prevalence of some Southern African countries, are also encouraging factors. The work of international NGOs in partnership with local organizations in these and other countries confirms that men will increasingly choose vasectomy under conducive conditions.

We also hope that a courageous and visionary donor in the international FP ecosystem, unbound by an imperative to demonstrate substantial immediate increases in method uptake or “cost-effectiveness,” might embrace this effort and fund it accordingly—that would be a true “innovation.” The falloffs in vasectomy uptake documented in Brazil and Bangladesh when donor funding for vasectomy was curtailed underscore the importance of sustained (and additive) funding. A related point regarding possible donor-funded development of new methods of vasectomy (surgical or nonsurgical) is that any new method would almost certainly face the same array of sociocultural impediments as the current vasectomy method. Programmatic needs—to ensure accurate understanding among clients and providers, foster demand, address gender constraints, and maintain adequate provider capacity—would also be the same.

Finally, we note that drivers of potential interest in vasectomy continue: the gender equality and women’s empowerment agenda is reflected in many government commitments to the health-related SDGs^76^; demand to limit further births is growing as desired family size and fertility fall in LMICs; social and mass media are ubiquitous, holding promise for increasing accurate knowledge about vasectomy and normalizing it as a method consideration; safe surgery is receiving more attention in development efforts; and in every region, culture, and country, some men already do choose vasectomy.

So let’s walk our talk on broadening method mix, enhancing constructive male engagement in FP and working toward gender equality by championing vasectomy and giving it greater attention, priority, and funding in LMICs. Doing so can help to reverse a decades-long, global downward trend and make this method a true, rights-based contraceptive option for more people.

## Supplementary Material

GHSP-D-22-00369-Supplement.pdf

## Notes

**Peer Reviewed**
