# Supplementary material for: Down But Not Out: Vasectomy Is Faring Poorly Almost Everywhere—We Can Do Better To Make It A True Method Option
Source: Glob Health Sci Pract. 2023 Feb 28;11(1):e2200369. doi: 10.9745/GHSP-D-22-00369 (PMC9972380; doi:10.9745/GHSP-D-22-00369)
Supplement: GHSP-D-22-00369-Supplement.pdf [file GHSP-D-22-00369-Supplement.pdf]

**Supplement to**: Jacobstein R, Radloff S, Khan F, et al. Down but not out: vasectomy is faring poorly almost everywhere—doing better to make it a true method option. *Glob Health Sci Pract*. 2023;11(1):e2200369.

# SUPPLEMENT TABLES OF COUNTRIES WITH VASECTOMY PREVALENCE BELOW 1%

Supplement Table 1. Countries in Eastern Africa and Southern Africa with Vasectomy Prevalence Below 1%

Supplement Table 2. Countries in Middle Africa and Western Africa With Vasectomy Prevalence Below 1%

Supplement Table 3. Countries in Northern Africa, Central Asia, and Western Asia With Vasectomy Prevalence Below 1%

Supplement Table 4. LMICs in Southern Asia, South-Eastern Asia and Eastern Asia With Vasectomy Prevalence Below 1%

Supplement Table 5. LMICs in LAC With Vasectomy Prevalence Below 1%

## Supplement Table 1. Countries in Eastern Africa and Southern Africa With Vasectomy Prevalence Below 1%

| Country | Survey End Year | Age Group | MCPR (%) | Vasectomy prevalence (%)* | Vasectomy method share* | Tubectomy prevalence (%)* |
|---|---|---|---|---|---|---|
| **Angola** | **2016** | **15-49** | **12.5** | **-** | **--** | **0.1** |
| | 2001 | 15-49 | 4.9 | 0.0 | 0.0 | 0.1 |
| **Burundi** | **2017** | **15-49** | **22.4** | **0.1** | **0.1%** | **0.5** |
| | 2009 | 15-49 | 19.6 | 0.0 | -- | -- |
| **Ethiopia** | **2019** | **15-49** | **35.8** | **--** | **--** | **0.3** |
| | 2011 | 15-49 | 27.3 | -- | -- | 0.5 |
| **Kenya** | **2020** | **15-49** | **60.1** | **0.0** | **--** | **2.9** |
| | 2009 | 15-49 | 39.4 | 0.1 | 0.2% | 2.4 |
| **Madagascar** | **2018** | **15-49** | **40.5** | **0.1** | **0.2%** | **0.5** |
| | 2009 | 15-49 | 29.2 | 0.1 | 0.3% | 1.1 |
| **Malawi** | **2020** | **15-49** | **64.7** | **0.1** | **0.2%** | **8.2** |
| | 2010 | 15-49 | 42.2 | 0.1 | 0.3% | 4.7 |
| **Mozambique** | **2015** | **15-49** | **25.3** | **0.0** | **--** | **0.2** |
| | 2008 | 15-49 | 14.7 | -- | -- | 0.2 |
| **Rwanda** | **2020** | **15-49** | **56.9** | **0.2** | **0.4%** | **2.0** |
| | 2011 | 15-49 | 44.5 | 0.0 | -- | 0.8 |
| **South Africa** | **2016** | **15-49** | **54.0** | **0.6** | **1.1%** | **7.7** |
| | 2004 | 15-49 | 59.8 | 0.7 | 1.2% | 14.3 |
| **South Sudan** | **2015** | **15-49** | **5.0** | **--** | **--** | **--** |
| | 2006 | 15-49 | 3.3 | -- | -- | 0.0 |
| **Uganda** | **2021** | **15-49** | **42.7** | **--** | **--** | **2.1** |
| | 2011 | 15-49 | 26.0 | 0.1 | 0.3% | 2.9 |
| **Tanzania** | **2016** | **15-49** | **32.0** | **0.1** | **0.3%** | **3.4** |
| | 2005 | 15-49 | 20.0 | 0.0 | -- | 2.6 |
| **Zambia** | **2018** | **15-49** | **47.5** | **--** | **--** | **1.5** |
| | 2007 | 15-49 | 32.7 | -- | -- | 1.9 |
| **Zimbabwe** | **2015** | **15-49** | **65.8** | **0.0** | **--** | **0.8** |
| | 2006 | 15-49 | 58.4 | 0.1 | 0.2% | 2.0 |

**Data Source:** UN Department of Economic and Social Affairs, Population Division (2022). *World Contraceptive Use 2022*. **Notes**: Data are for women married or in union (MWRA). Vasectomy's method share is its proportion of use among MWRA users of modern contraception. Lesotho and Namibia do not meet study criteria for population size, but have vasectomy prevalence of 0.4% (2018) and 0.3% (2013) respectively, with tubectomy prevalence of 1.7% and 6.4% respectively. *Dashes in these columns indicate that a value for vasectomy prevalence or tubectomy prevalence was not provided in the country's survey report (due to the very low number of surveyed women indicating reliance on vasectomy.)

**Supplement to**: Jacobstein R, Radloff S, Khan F, et al. Down but not out: vasectomy is faring poorly almost everywhere—doing better to make it a true method option. *Glob Health Sci Pract*. 2023;11(1):e2200369.

## Supplement Table 2. Countries in Middle Africa and Western Africa With Vasectomy Prevalence Below 1%

| Country | Survey End Year | Age Group | MCPR (%) | Vasectomy prevalence (%)* | Vasectomy method share* | Tubectomy prevalence (%)* |
|---|---|---|---|---|---|---|
| **Benin** | **2018** | **15-49** | **12.0** | **--** | **--** | **--** |
| | 2006 | 15-49 | 6.1 | -- | -- | 0.3 |
| **Burkina Faso** | **2020** | **15-49** | **28.1** | **--** | **--** | **0.2** |
| | 2011 | 15-49 | 15.0 | -- | -- | 0.2 |
| **Cameroon** | **2018** | **15-49** | **15.0** | **--** | **--** | **0.3** |
| | 2006 | 15-49 | 15.2 | -- | -- | 0.4 |
| **Cent. Afr. Rep.** | **2019** | **15-49** | **14.4** | **0.1** | **0.7%** | **0.2** |
| | 2006 | 15-49 | 11.2 | 0.1 | 0.9% | 0.3 |
| **Chad** | **2019** | **15-49** | **6.7** | **0.0** | **--** | **0.1** |
| | 2010 | 15-49 | 4.5 | -- | -- | 0.1 |
| **Congo** | **2015** | **15-49** | **18.5** | **0.0** | **--** | **0.2** |
| | 2005 | 15-49 | 12.7 | -- | -- | 0.2 |
| **Cote d'Ivoire** | **2017** | **15-49** | **18.9** | **0.0** | **--** | **0.2** |
| | 2006 | 15-49 | 9.3 | -- | -- | -- |
| **DR Congo** | **2018** | **15-49** | **17.6** | **0.0** | **--** | **0.6** |
| | 2007 | 15-49 | 5.8 | -- | -- | 0.8 |
| **Ghana** | **2018** | **15-49** | **24.3** | **0.0** | **--** | **1.7** |
| | 2008 | 15-49 | 16.6 | -- | -- | 1.6 |
| **Guinea** | **2018** | **15-49** | **10.3** | **0.0** | **--** | **0.2** |
| | 2012 | 15-49 | 4.6 | -- | -- | 0.1 |
| **Liberia** | **2020** | **15-49** | **23.8** | **--** | **--** | **0.2** |
| | 2013 | 15-49 | 19.1 | -- | -- | 0.3 |
| **Mali** | **2018** | **15-49** | **16.4** | **--** | **--** | **0.4** |
| | 2010 | 15-49 | 8.8 | -- | -- | -- |
| **Mauritania** | **2020** | **15-49** | **10.0** | **--** | **--** | **--** |
| | 2011 | 15-49 | 10.1 | 0.2 | 2.0% | 0.2 |
| **Niger** | **2021** | **15-49** | **10.0** | **--** | **--** | **0.0** |
| | 2012 | 15-49 | 12.2 | -- | -- | 0.1 |
| **Nigeria** | **2018** | **15-49** | **18.8** | **0.0** | **--** | **0.5** |
| | 2008 | 15-49 | 9.7 | -- | -- | 0.4 |
| **Senegal** | **2019** | **`15-49** | **25.4** | **0.0** | **--** | **0.7** |
| | 2011 | 15-49 | 12.1 | -- | -- | 0.2 |
| **Sierra Leone** | **2019** | **15-49** | **14.4** | **0.1** | **0.7%** | **0.2** |
| | 2010 | 15-49 | 10.0 | -- | -- | -- |
| **Togo** | **2017** | **15-49** | **21.5** | **0.1** | **0.5%** | **1.0** |
| | 2006 | 15-49 | 11.6 | 0.0 | -- | 0.3 |

**Data Source:** UN Department of Economic and Social Affairs, Population Division (2022). *World Contraceptive Use 2022*. **Notes**: Data are for women married or in union (MWRA). Vasectomy's method share is its proportion of use among MWRA users of modern contraception. *Dashes in these columns indicate that a value for vasectomy prevalence or tubectomy prevalence was not provided in the country's survey report (due to the very low number of surveyed women indicating reliance on vasectomy.)

**Supplement to**: Jacobstein R, Radloff S, Khan F, et al. Down but not out: vasectomy is faring poorly almost everywhere—doing better to make it a true method option. *Glob Health Sci Pract*. 2023;11(1):e2200369.

## Supplement Table 3. Countries in Northern Africa, Central Asia, and Western Asia With Vasectomy Prevalence Below 1%

| Country | Survey End Year | Age Group | MCPR (%) | Vasectomy prevalence (%)* | Vasectomy method share* | Tubectomy prevalence (%)* |
|---|---|---|---|---|---|---|
| **Algeria** | **2019** | **15-49** | **44.9** | **0.1** | **0.2%** | **0.4** |
| | 2012 | 15-49 | 49.5 | 0.0 | -- | 0.5 |
| **Azerbaijan** | **2011** | **15-49** | **13.9** | **--** | **--** | **0.9** |
| | 2001 | 15-44 | 11.9 | -- | -- | 1.2 |
| **Egypt** | **2014** | **15-49** | **56.9** | **--** | **--** | **1.2** |
| | 2005 | 15-49 | 56.5 | -- | -- | 1.3 |
| **Jordan** | **2018** | **15-49** | **37.4** | **--** | **--** | **1.5** |
| | 2009 | 15-49 | 42.0 | 0.0 | -- | 2.6 |
| **Kazakhstan** | **2018** | **15-49** | **50.1** | **0.0** | **--** | **1.3** |
| | 2011 | 15-49 | 49.8 | 0.0 | -- | 1.2 |
| **Kyrgyzstan** | **2018** | **15-49** | **37.8** | **0.2** | **0.5%** | **1.2** |
| | 2012 | 15-49 | 33.7 | -- | -- | 1.6 |
| **Morocco** | **2018** | **15-49** | **59.1** | **0.0** | **--** | **1.0** |
| | 2011 | 15-49 | 58.6 | -- | -- | -- |
| **Saudi Arabia** | **2019** | **15-49** | **24.4** | **0.1** | **0.4%** | **--** |
| | 2007 | 15-49 | 23.8 | -- | -- | -- |
| **State of Palestine** | **2020** | **15-49** | **42.8** | **0.3** | **0.7%** | **1.8** |
| | 2010 | 15-49 | 44.2 | 0.0 | -- | 2.6 |
| **Sudan** | **2014** | **15-49** | **11.7** | **--** | **--** | **--** |
| | 2006 | 15-49 | 9.0 | -- | -- | 0.5 |
| **Tajikistan** | **2017** | **15-49** | **27.1** | **--** | **--** | **0.8** |
| | 2007 | 15-49 | 31.7 | -- | -- | -- |
| **Tunisia** | **2018** | **15-49** | **44.3** | **0.1** | **0.2%** | **1.3** |
| | 2012 | 15-49 | 50.9 | 0.0 | -- | 1.1 |
| **Turkmenistan** | **2019** | **15-49** | **47.3** | **0.0** | **--** | **0.3** |
| | 2006 | 15-49 | 46.9 | -- | -- | -- |
| **Turkey** | **2018** | **15-49** | **49.0** | **--** | **--** | **10.4** |
| | 2008 | 15-49 | 46.0 | 0.1 | 0.2% | 8.3 |
| **Yemen** | **2013** | **15-49** | **25.2** | **0.1** | **0.4%** | **2.3** |
| | 2006 | 15-49 | 19.2 | -- | -- | 2.3 |

**Data Source:** UN Department of Economic and Social Affairs, Population Division (2021). *World Contraceptive Use 2022.* **Notes**: Data for women married or in union (MWRA). Vasectomy's method share is its proportion of use among MWRA users of modern contraception. For Qatar, see Table 1. Oman does not meet study criteria for population size, but has vasectomy prevalence of 0.2% and tubectomy prevalence of 3.4% (2014). *Dashes in these columns indicate that a value for vasectomy prevalence or tubectomy prevalence was not provided in the country's survey report (due to the very low number of surveyed women indicating reliance on vasectomy.)

**Supplement Table 4. Countries in Southern Asia, South-Eastern Asia and Eastern Asia With Vasectomy Prevalence Below 1%**

| Country | Survey End Year | Age Group | MCPR (%) | Vasectomy prevalence (%)* | Vasectomy method share* | Tubectomy prevalence (%)* |
|---|---|---|---|---|---|---|
| **Afghanistan** | **2018** | **12-49** | **17.4** | **--** | **--** | **0.2** |
| | 2010 | 12-49 | 19.9 | 0.0 | -- | 1.4 |
| **Bangladesh** | **2019** | **15-49** | **59.1** | **0.5** | **0.8%** | **3.3** |
| | 2011 | 15-49 | 52.1 | 1.2 | 2.3% | 5.0 |
| **Cambodia** | **2014** | **15-49** | **38.8** | **0.1** | **0.3%** | **3.0** |
| | 2006 | 15-49 | 27.2 | 0.1 | 0.4% | 1.7 |
| **India** | **2016** | **15-49** | **47.8** | **0.3** | **0.6%** | **36.0** |
| | 2006 | 15-49 | 48.6 | 1.0 | 2.1% | 37.3 |
| **Indonesia** | **2018** | **15-49** | **54.2** | **0.3** | **0.6%** | **1.9** |
| | 2008 | 15-49 | 55.2 | 0.4 | 0.7% | 1.2 |
| **Iraq** | **2018** | **15-49** | **36.1** | **0.1** | **0.3%** | **3.0** |
| | 2011 | 15-49 | 33.8 | -- | -- | 3.1 |
| **Laos** | **2017** | **15-49** | **49.0** | **0.0** | **--** | **4.4** |
| | 2005 | 15-49 | 35.0 | 0.0 | -- | 4.7 |
| **Malaysia** | **2014** | **15-49** | **34.3** | **--** | **--** | **6.9** |
| | 2004 | 15-49 | 32.3 | -- | -- | -- |
| **Myanmar** | **2016** | **15-49** | **51.3** | **0.3** | **0.6%** | **4.8** |
| | 2007 | 15-49 | 38.4 | 1.0 | 2.6% | 4.4 |
| **Pakistan** | **2019** | **15-49** | **23.4** | **--** | **--** | **7.8** |
| | 2008 | 15-49 | 19.3 | -- | -- | 6.5 |
| **Papua New Guinea** | **2018** | **15-49** | **30.5** | **0.8** | **2.6%** | **8.0** |
| | 2007 | 15-49 | 24.3 | 0.5 | 2.1% | 8.6 |
| **Philippines** | **2017** | **15-49** | **40.1** | **0.0** | **--** | **7.4** |
| | 2006 | 15-49 | 35.9 | 0.1 | 0.3% | 10.4 |
| **Sri Lanka** | **2016** | **15-49** | **53.6** | **0.0** | **--** | **14.0** |
| | 2007 | 15-49 | 52.5 | 0.7 | 1.3% | 16.3 |
| **Thailand** | **2019** | **15-49** | **71.3** | **0.3** | **0.4%** | **25.2** |
| | 2009 | 15-49 | 77.5 | 0.9 | 1.2% | 23.7 |
| **Viet Nam** | **2019** | **15-49** | **66.5** | **0.1** | **0.2%** | **1.1** |
| | 2010 | 15-49 | 67.5 | 0.2 | 0.3% | 3.0 |

**Data Source:** UN Department of Economic and Social Affairs, Population Division (2021). *World Contraceptive Use 2022*. **Notes**: Data for women married or in union (MWRA). Vasectomy's method share is its proportion of use among MWRA users of modern contraception. For Bhutan, China, Iran, Nepal, and Taiwan see Table 1. *Dashes in these columns indicate that a value for vasectomy prevalence or tubectomy prevalence was not provided in the country's survey report (due to the very low number of surveyed women indicating reliance on vasectomy.)

## Supplement Table 5. LMICs in LAC With Vasectomy Prevalence Below 1%

| Country | Survey End Year | Age Group | MCPR (%) | Vasectomy prevalence (%)* | Vasectomy method share* | Tubectomy prevalence (%) |
|---|---|---|---|---|---|---|
| **Argentina** | **2020** | **15-49** | **68.2** | **0.2** | **0.3%** | **15.7** |
| | 2013 | 15-49 | 78.2 | 0.0 | -- | 5.6 |
| **Bolivia** | **2016** | **15-49** | **45.1** | **0.1** | **0.2%** | **9.7** |
| | 2008 | 15-49 | 34.5 | 0.1 | 0.3% | 6.5 |
| **Cuba** | **2019** | **15-49** | **67.9** | **0.0** | **--** | **20.7** |
| | 2010 | 15-49 | 73.2 | 0.0 | -- | 23.9 |
| **Dominican Republic** | **2019** | **15-49** | **62.0** | **0.1** | **0.2%** | **30.5** |
| | 2010 | 15-49 | 71.1 | -- | -- | 44.1 |
| **Ecuador** | **2019** | **15-49** | **72.4** | **0.4** | **0.5%** | **28.8** |
| | 2012 | 15-49 | 71.7 | 0.3 | 0.4% | 32.3 |
| **El Salvador** | **2014** | **15-44** | **67.6** | **0.3** | **0.4%** | **30.8** |
| | 2008 | 15-49 | 66.1 | 0.3 | 0.5% | 35.3 |
| **Guatemala** | **2015** | **15-49** | **48.9** | **0.6** | **1.2%** | **21.0** |
| | 2009 | 15-49 | 44.0 | 0.8 | 1.8% | 18.9 |
| **Haiti** | **2017** | **15-49** | **31.8** | **0.2** | **0.6%** | **1.2** |
| | 2006 | 15-49 | 24.8 | -- | -- | 2.1 |
| **Honduras** | **2012** | **15-49** | **63.8** | **0.3** | **0.5%** | **22.3** |
| | 2006 | 15-49 | 56.3 | 0.3 | 0.5% | 21.2 |
| **Nicaragua** | **2012** | **15-49** | **77.4** | **0.4** | **0.5%** | **29.8** |
| | 2007 | 15-49 | 69.8 | 0.4 | 0.6% | 24.3 |
| **Panama** | **2015** | **15-49** | **46.9** | **0.4** | **0.9%** | **16.8** |
| | 2009 | 15-49 | 59.5 | 0.2 | 0.3% | 27.8 |
| **Paraguay** | **2016** | **15-49** | **66.5** | **0.1** | **0.2%** | **8.8** |
| | 2008 | 15-49 | 70.7 | 0.2 | 0.3% | 9.9 |
| **Peru** | **2018** | **15-49** | **55.0** | **0.4** | **0.7%** | **9.6** |
| | 2008 | 15-49 | 49.1 | 0.3 | 0.6% | 9.8 |

**Data Source:** UN Department of Economic and Social Affairs, Population Division (2022). *World Contraceptive Use 2022.* **Notes:** Data for women married or in union (MWRA). Vasectomy's method share is its proportion of use among MWRA users of modern contraception. For Brazil, Colombia, Costa Rica and Mexico, see Table 1. Uruguay does not meet study criteria for population size, but had vasectomy prevalence of 0.6% and tubectomy prevalence of 9.1% in 2015, and 0.8% and 5.4% in 2004. *Dashes in these columns indicate that a value for vasectomy prevalence was not provided in the country's survey report (due to the very low number of surveyed women indicating reliance on vasectomy.)
